# Ultraelastic and High‐Conductivity Multiphase Conductor with Universally Autonomous Self‐Healing

**DOI:** 10.1002/advs.202205485

**Published:** 2022-11-09

**Authors:** Jarkko Tolvanen, Mikko Nelo, Heidi Alasmäki, Tuomo Siponkoski, Piia Mäkelä, Timo Vahera, Jari Hannu, Jari Juuti, Heli Jantunen

**Affiliations:** ^1^ Microelectronics Research Unit Faculty of Information Technology and Electrical Engineering University of Oulu P.O. Box 4500 Oulu FI‐90014 Finland; ^2^ Research Unit of Medical Imaging Physics and Technology Faculty of Medicine University of Oulu P.O. Box 5000 Oulu FI‐90014 Finland

**Keywords:** organic conductors, poly(3,4‐ethylenedioxythiophene):poly(styrenesulfonate), self‐healing, soft sensors, stretchable electronics

## Abstract

Next‐generation, truly soft, and stretchable electronic circuits with material level self‐healing functionality require high‐performance solution‐processable organic conductors capable of autonomously self‐healing without external intervention. A persistent challenge is to achieve required performance level as electrical, mechanical, and self‐healing properties optimized in tandem are difficult to attain. Here heterogenous multiphase conductor with cocontinuous morphology and macroscale phase separation for ultrafast universally autonomous self‐healing with full recovery of pristine tensile and electrical properties in less than 120 and 900 s, respectively, is reported. The multiphase conductor is insensitive to flaws under stretching and achieves a synergistic combination of conductivity up to ≈1.5 S cm^−1^, stress at break ≈4 MPa, toughness up to >81 MJ m^−3^, and elastic recovery exceeding 2000% strain. Such properties are difficult to achieve simultaneously with any other type of material so far. The solution‐processable multiphase conductor offers a paradigm shift for damage tolerant and environmentally resistant soft electronic components and circuits with material level self‐healing.

## Graphical abstract

Ultraelastic and high‐conductivity organic conductor–elastomer blend with ultrafast and efficient self‐healing in universal conditions is demonstrated. The notch‐insensitive multiphase conductor, with a cocontinuous morphology and controllable heterogeneity, achieves synergistically optimized properties including toughness >80 MJ m^−3^, stress at break >4 MPa, elastic recovery >2000% strains, and conductivity up to ≈1.5 S cm^−1^.

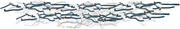

## 1 Introduction

Solution‐processable organic conductors have a fundamental role for large‐scale production of polymer‐based flexible electronics with multilayered component or device architectures.^[^ ^1^, ^2^, ^3^ ^]^ Electronic properties of amorphous or semicrystalline conjugated polymers and small molecule semiconductors can be easily tailored, and their mechanical compliances matched to that of biological systems through innovative molecular designs and multicomponent approaches^.[^ ^3^, ^4^, ^5^, ^6^, ^7^, ^8^ ^]^ These materials are in a key role to achieve imperceptible skin‐interfaced sensing and communications systems, implantable bioelectronics, or prosthetics with integration of smart skins and neuromorphic devices leading to future technological advances.^[^ ^4^, ^5^, ^6^, ^9^ ^]^ However, several material level challenges remain for highly deformable and damage tolerant organic electronics that have excellent electromechanical performance. A major challenge has been achieving high levels of elasticity and fast autonomous self‐healability with the use of stiff and low molecular mobility polymer chains that can easily crack under extremely large deformations.^[^ ^4^, ^8^ ^]^ At the same time, challenges with mechanical and morphological stability exist in the materials that often result in a failure of deformable electronic component or device performance degradation over time.^[^ ^9^, ^10^ ^]^

On the other hand, it is possible to solve many of these challenges simultaneously at the material level. For instance, a promising material design strategy could be to have a phase‐separated interpenetrated network with a coexistence of at least two continuous structures (i.e., cocontinuous morphology) and controllable heterogeneity. Heterogenous phase‐separated morphologies are already known not only to be important to achieve mechanically robust self‐healing elastomers, with high molecular mobility, but also for improving stability and electromechanical performance of nonhealable semiconductor–elastomer blends.^[^ ^9^, ^11^, ^12^, ^13^, ^14^, ^15^, ^16^, ^17^ ^]^ Regardless, it has been yet difficult to introduce electrical functionalities into the above‐mentioned self‐healing materials while improving, or without compromising, the tensile and/or self‐healing properties due to mutual exclusiveness of the characteristics.

High‐performance soft conductors with autonomous self‐healing have paramount importance for damage tolerant and puncture resistant organic electronics components and circuits. Hence, numerous alternative design strategies for self‐healing electronic materials have been proposed based on designing stretchable conductors and deformable electronics components. These include blending conductive fillers such as high‐aspect ratio nanomaterials,^[^ ^18^ ^]^ (semi)conducting polymers,^[^ ^19^, ^20^ ^]^ inorganic salts or electrolytes,^[^ ^21^, ^22^, ^23^ ^]^ or ionic liquids^[^ ^24^, ^25^, ^26^, ^27^ ^]^ into self‐healing matrices made of synthetic rubber‐like materials, gels, or hydrogels. These approaches have enabled autonomously self‐repairing conductors. However, achieving efficient self‐healing after repeated damages for tensile and electrical properties has been difficult. One of the challenges is that the interfaces often repair only between the macromolecules, having certain chemical compositions, and not between the actual fillers present in the polymer matrix. Hence, the elastomeric networks, with presence of immobile and high modulus fillers, can be vulnerable to crack growth and propagation by also considering the partial self‐healability and flaw sensitive nature of many single elastomeric networks. Alternatively, 1D nanomaterials have been deposited onto self‐healing elastomers^[^ ^28^, ^29^, ^30^ ^]^ to form a prestrained out‐of‐the‐plane geometries, or by directly using a liquid‐metal based electrical wiring.^[^ ^31^, ^32^, ^33^ ^]^ However, stretchable out‐of‐plane structures are even more difficult to self‐repair once physically broken as precise alignment of layers is even more cumbersome and the interfaces inside the conductive layers are unable to repair. On the other hand, liquid metals have limited operating temperatures for stretchable autonomously self‐healing conductors due to their liquid–solid transition temperature.

Several material level trade‐offs and compromises must be made in terms of tensile, electrical, and self‐healing properties with any of the above‐mentioned strategies. This has resulted in a moderate overall performance so far. Several approaches, with either improved electrical and mechanical properties, have still restricted environmental resistance in terms of self‐healability, and they may only partially recover their pristine tensile and electrical properties after mechanical damages. One of the reasons for imperfect self‐healability is often their covalently bonded electrical percolation networks which have low adhesion at the interfacial regions to the self‐healing matrix,^[^ ^30^, ^34^, ^35^ ^]^ thus restrict the mechanical integrity of the stretchable conductors under large deformations.

Herein, we report a novel flaw insensitive organic conductor–elastomer blend that is also capable of self‐healing in challenging environmental conditions including low or high pressures and temperatures, underwater, saline, acidic or alkaline conditions. The multiphase conductor, with heterogenous cocontinuous morphology, achieves ultrafast recovery of pristine tensile and electrical properties after short self‐healing times when mechanically damaged. Interestingly, the vertically phase‐separated interpenetrated network allows to synergistically optimize the mechanical, electrical, and self‐healing properties when further controlling the heterogeneity, kinetics of phase separation and structure formation. We propose that the material design concept is applicable, with further work, to many other organic conductor–elastomer blends to not only increase their overall performance but to render them capable of self‐healing without external intervention.

## 2 Results

### 2.1 Multiphase Material Design

**Figure 1:**
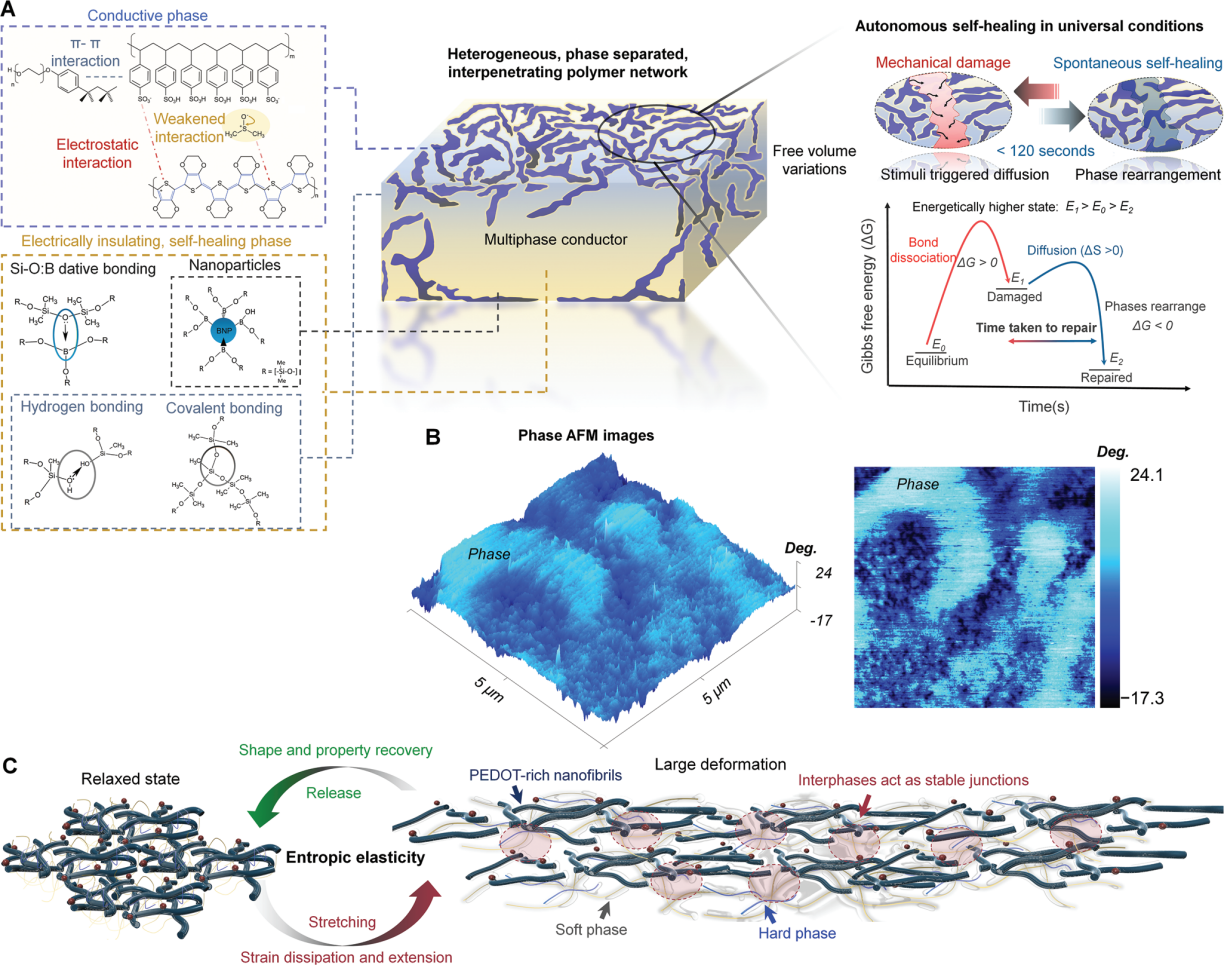
Schematic illustration of the multiphase conductor design. a) Organic conductor–elastomer blend with autonomous chemico‐physical self‐repair in universal conditions. Upon mechanical damage, or a deformation, bond dissociation and displacement of macromolecular segments leads to transition into energetically higher state (*E*_1_) from the equilibrium state (*E*_0_). Thus, Gibbs free energy (Δ*G*) of the multiphase conductor changes upon spontaneous self‐healing (assuming Δ*G =* −*T*Δ*S*). Then, energetically higher state becomes deactivated as wetting by polymers occurs (i.e., transition from *E*_1_ to *E*_2_ occurs). This leads to simultaneously occurring rebonding reactions and movement of macromolecular segments (entropy change (Δ*S)*  > 0) to recover the equilibrium state again (*E*_2_). b) 2D and 3D phase atomic force microscopy (AFM) images of the multiphase conductor. c) Schematic illustration of the entropy elasticity driven by the hydrophobic and hydrophilic interactions. The interphases act as a stable junction points even extremely large deformations which enables shape and property recovery after release of stress.

A schematic illustration of the electrically conductive multiphase conductor, with autonomous self‐healing and entropy‐driven elasticity, is shown in **Figure** **1**a–c. It is a well‐known that organic conductor–elastomer blend would require a well‐defined phase separation, between the electrically active and nonactive phases, and a long‐range molecular order in the *π*‐conjugated polymer to achieve an efficient charge transport for a good conductivity.^[^ ^11^ ^]^ Hence, we rationalized that a vertically phase‐separated interpenetrated network, with a cocontinuous multiphase material, would be an ideal way for achieving a soft conductor with synergistically optimized electrical, mechanical, and self‐healing properties. In our previous work, we optimized tensile and self‐healing properties of electrically nonconductive siloxane‐based elastomer (Figure S1, Supporting Information) with bimodal polymer chain length distribution, heterogeneity, and macroscale phase separation.^[^ ^13^ ^]^ By forming a multicomponent blend, where kinetics of phase separation and structure formation could be further controlled, it would be possible to introduce electrical conductivity while considerably further improve the excellent tensile and self‐healing properties of the supramolecular elastomer. This would be possible, for example, with the use of poly(3,4‐ethylenedioxythiophene):poly(styrenesulfonate) (PEDOT:PSS) which is one most well‐known *π*‐conjugated polymers due to numerous applications in soft electronics.

On the basis of recoupling lattice model, the localized variations of glass transition temperatures, in a heterogenous self‐healing multiphase material, would be energetically and spatially favorable for the localized entropy changes (Δ*S*) and available free volume near the damaged site. High flexibility of the polymer chains would be prerequisite for a large number of possible chain configurations for the free and tethered chains to expand into void caused by a rupture. A higher flexibility would result in larger change of a Gibbs free energy (Δ*G*).^[^ ^36^, ^37^ ^]^ In such multiphase material, the varied segmental mobility of macromolecules present in the material system would increase due to volume expansion gradients. The reason is that lesser spatial constraints and friction would exist between the polymer chains within the polymer lattice. Hence, existing microlevel variations and high free volume would result in a faster rebonding and macroscopic network rearrangements through the hydrophilic and the hydrophobic interactions. The heterogeneity and large domain sizes present in the material would restrict the nonreversible flow in the elastomeric network even at extremely large deformations,^[^ ^13^ ^]^ thus allow entropic elasticity (as shown in Figure 1c).

The self‐healing mechanism of the multiphase material, consisting of organic conductor–elastomer blend, can be ascribed from the thermodynamics point of view.^[^ ^36^, ^37^, ^38^ ^]^ When the autonomous self‐healing material is at a phase equilibrium, it would be thermodynamically at the lowest energy state (either *E*_0_ or *E*_2_) when at relaxed state (Δ*G* = 0). This reason is that the entropy and enthalpy changes are both zero when pressure and temperature would also remain constant. The multiphase material would be then able to transition from the equilibrium (*E*_0_) into a metastable state (*E*_1_) through reversible bond dissociations and displacements of polymer chain segments by the introduced stimuli‐responsiveness. This would result in a localized ΔS in the material system (assuming Δ*G =* −*T*Δ*S*, when Δ*S >* 0).^[^ ^36^, ^37^ ^]^ Hence, a spontaneous shift into energetically distinct state would occur while the localized volume would increase near the damage site due to lower glass transition temperature (Figure 1a). In this particular case, the transitions between the steady state and nonequilibrium states would be possible through embedded dipole–dipole interactions present in the self‐healing supramolecular elastomer. The transition would occur when at spatially favorable physical distance to initiate wetting by the polymer. This would facilitate a self‐healing that is reliant on both the chemical interactions and physical elastomeric network properties (i.e., chemico‐physical process). The self‐healing would be then driven by both interfacial and entropic energy, and existing volume expansion gradients with the consideration that the rebonding reactions would parallel with the positive conformational entropy changes (Δ*S >* 0; as previously discussed^[^ ^37^, ^38^, ^39^ ^]^). The enthalpic term (Δ*H*) would be of consideration only once the polymer chains would approach their Gaussian state. However, such material systems would then require a delicate energetical balance for maintaining phase equilbria over time which would be a prerequisite for the long‐term morphologically stability (as shown later on).

### 2.2 Formation of Nanofibrils and Blending

To achieve a good conductivity, with pristine PEDOT:PSS, it is necessary to induce a morphological change with a conductivity enhancer. This can be accomplished by the formation of PEDOT‐rich nanofibrils with the use of a polar solvent. The main function of the polar solvent would be then to weaken the electrostatic interaction between PEDOT and PSS chain segments.^[^ ^40^, ^41^ ^]^ In this particular case, we have chosen a dimethyl sulfoxide (DMSO), which is a well‐known conductivity enhancer and plasticizer for PEDOT:PSS. The use of DMSO is known to facilitate a conformational and a morphological change by coalescing the PEDOT‐rich cores of PEDOT:PSS microgel particles that are present in the aqueous solution of PEDOT:PSS. As a consequence of the promoted phase separation, between the PEDOT and the PSS chain segments, the *π*–*π* stacking distance further reduces through a high degree molecular packing.^[^ ^41^, ^42^, ^43^ ^]^

The PEDOT‐rich nanofibril network is not only important in terms of the conductivity, but to allow distinct macroscopic deformation modes (e.g., slipping and axial rotation) for the stiff and lower molecular mobility polymer chain segments. The strong immiscibility between the rod segments (PEDOT:PSS) and the coil segments (soft and hard phase), present in the multiphase conductor, supposedly leads to a repulsion which would improve phase rearrangements due to strong hydrophilic and hydrophobic interactions. We suppose this could result in a high segmental mobility and reduce spatial stress concentrations (in the phase‐separated interpenetrated network) and in turn, result in a very effective autonomous self‐healability and strain energy dissipation upon deformations, respectively.

Poor miscibility between the aqueous PEDOT:PSS and bimodal self‐healing elastomer mainly arises from the swollen microgel particles and *π*–*π* stacking. Thus, an amphiphilic surfactant was required to form a homogeneous blend with PEDOT:PSS during the blending of the components. We suppose that other type of organic conductors may not actually require surfactant to prepare vertically phase‐separated morphologies with desirable domain sizes. Hence, morphology and structure of multiphase conductor would be then largely influenced by the choice of solvent, solubility parameters, and processing conditions.^[^ ^9^ ^]^ In this case, we have chosen a polyethylene glycol tert‐octylphenyl ether (i.e., Triton X‐100) that is known to enable a dispersion between hydrophobic and hydrophilic chain segments through absorption to the PSS benzene rings by *π*–*π* interactions and hydrogen bonding.^[^ ^44^, ^45^ ^]^ It should be noted that the choice of solvent and/or surfactant are solely dependent on the type of multicomponent blend and processing conditions required to prepare a desirable heterogenous cocontinuous morphology.^[^ ^9^, ^10^ ^]^ This leaves some form of design freedom, in terms of preparation of the self‐healing multiphase material, and further tuning the electromechanical and self‐healing properties.

### 2.3 Morphology and Structure Formation

A phase separation is a common phenomenon that occurs in many type of organic conductor–elastomer and donor–acceptor blends.^[^ ^9^, ^10^ ^]^ However, controlling the phase separation and phase coarsening can be cumbersome in such materials. In this particular case, the multicomponent blend undergoes a complex phase separation that relies on several compositional parameters, physiochemical interactions, and processing conditions (please see the Supporting Information) during the simultaneous and independent polymerization of thermodynamically incompatible phases (Figure S2, Supporting Information). The multiphase blend primary consists of three phases: a bimodal siloxane‐based self‐healing (with a soft and a hard phase^[^ ^13^ ^]^) and a PEDOT:PSS with a conductivity enhancer (Figure S1, Supporting Information). During solidification, the less soluble components are becoming concentrated at the solute–substrate interface as the concentration of the solute increases. Due to the concentration dependency on the value of Gibbs free energy of mixing, the phase separation increased with decreasing the insulating to conducting phase ratios and with the weight of the first solution. One of the main reasons was the use of aqueous solution of PEDOT:PSS with extremely low solid volume content (≈1.3 wt%). Because mixing time of third solution influences the entropy of mixing, we found that the longer mixing times resulted in poorer phase separation.

The optical images and phase atomic force microscopy (AFM) images show heterogeneous and cocontinuous multiphase conductors (Figures S3–S8, Supporting Information). The phase separation and phase coarsening were found to be highly dependent on both multiphase conductor and bimodal self‐healing elastomer compositions. The heterogeneity, domain sizes, and sharpness of the domain edges vary with several compositional parameters (Figures S5–S7, Supporting Information). As shown, the PEDOT‐rich islands (darker or blue/purple) were surrounded by the soft and hard phase domains of the bimodal self‐healing elastomer (brighter or orange/green). The PEDOT‐rich and hard phase‐rich domains tend to form a dual‐rich regions to the lateral direction on surface favoring flocculation of PEDOT‐rich microgel particles. Transition from a microdroplet morphology to a flocculated PEDOT‐rich microgel particles occurred as DMSO content increases (Figure S5, Supporting Information) over the critical threshold concentration. This was independent of the other compositional parameters or processing conditions. The increasing DMSO content also decreases domain sizes, and the domain edges were becoming more definable. By further increasing the DMSO content over the threshold concentration, the multiphase conductors become increasingly more heterogenous, in comparison, close to, or below the critical threshold (Figure S3, Supporting Information). The insulating to conduction phase ratios influence the heterogeneity (at moderate to high iratios) while well‐defined topological structures form as the phase separation and domain sizes decrease. By controlling the weight of the first solution, the phase separation increases to a certain degree and the domain edges are becoming sharper. As shown, the heterogeneity can be further controlled with the amount of amphiphilic surfactant that was capable to increase the interfacial adhesion.

### 2.4 Material Characterization

The spectral features shown by Fourier‐transform infrared spectroscopy (FTIR) confirm the successful synthesis of the multicomponent blend (Figure S9, Supporting Information). Absence of band intensities for the Si:O—B dative bonded absorption peak intensity (at wavenumber of 1340 cm^−1^), nonhydrogen (at 3700 cm^−1^), and intermolecular hydrogen bonded bands (at 3290 cm^−1^) indicate that the condensation reaction was complete.^[^ ^13^ ^]^ Stretching vibrations of thiophene rings were found at 670 cm^−1^ (for C—S—C bond), 1390 cm^−1^, and 1550 cm^−1^. Stretching vibrations for sulfonyl groups were not visible due to strong band intensity of Si(CH_3_)_2_ (at 1020–1090 cm^−1^) and Si(CH_3_)_2_—O—Si(CH_3_)_2_ (at 1260 cm^−1^).^[^ ^13^ ^]^ The band intensity of methyl group was visible at 2910 cm^−1^, which indicates that a residual DMSO exist in the conductor after the solidification. Wide‐band intensities appeared at wavenumber of 3290 cm^−1^ with a volume concentration of DMSO increasing. This was due to plasticizing effect of DMSO, which also results in morphological and structural change to occur in the multiphase conductor. The activation energy extracted from the ultraviolet‐visible‐near infrared spectra was found to be 3.95 eV (Figure S10a, Supporting Information). The value was lower than that for pristine PEDOT:PSS (≈5.0 eV). The absorption peak with the maximum at wavelenght of ≈940 nm further suggests the conformational change by the DMSO and the polaron delocalization (Figure S10b, Supporting Information).

Investigation of molecular interactions with temperature dependent FTIR spectra indicates an excellent thermal stability. No visible band intensity changes or shifts were found during the heating that relate to stretching vibrations of methyl group, intermolecular hydrogen bonds, or dative bonding. The thermogravimetric analysis revealed that the decomposition started at ≈280 °C due to presence of thiophene ring (Figure S11a, Supporting Information). The differential scanning calorimetry shows endothermic peaks close to the decomposition temperature of thiophene ring (Figure S11b, Supporting Information). The weight loss was negligible (less than 0.5%) up to ≈250 °C that further supports the thermal stability of the multiphase conductors. However, it should be noted that band intensity changes were found at wavenumbers of 1550 cm^−1^ (for stretching vibration of a thiophene ring) and in 1930–2370 cm^−1^. These band intensity changes indicate that reversible structural changes are occurring in the conductor during the heating (as shown later on).

Optical transmittances for individual films (with thicknesses of ≈25 µm) were ≈74% and ≈53% at 550 nm wavelength on glass and polyethylene terephthalate (PET), respectively (Figure S12a, Supporting Information). Due to obvious reasons, the optical transmittance would be significantly lower once the film thickness would increase. However, multiphase conductors would fulfill the requirement for optoelectronic and smart skin applications considering that the transparency would further increase with the use thin films. Therefore, the solution‐processable multiphase conductor shows considerable possibilities for preparation of self‐healing, transparent, and electrically conducting coatings on various substrates. This can be accomplished with any scalable and cost‐efficient printed electronics manufacturing methods, such as tape casting (Figure S12b, Supporting Information). However, it should be noted that the compositional dependent viscosity should be precisely adjusted for a specific printing method to achieve a desirable printing quality.

### 2.5 Electrical Properties and Anisotropy

**Figure 2:**
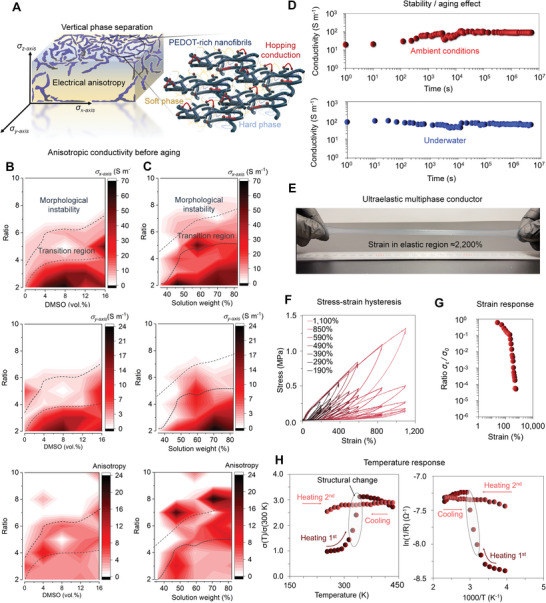
Electrical properties and stability. a) Schematic illustration of anisotropic conductivity and the corresponding measurement directions. b) Electrical conductivity parallel and perpendicular to the stencil printing direction of nanofibrils, and anisotropy (i.e., *σ_x_*_‐axis_/*σ_y_*_‐axis_) as a function of DMSO content and insulating to conducting phase ratios. The weight of the first solution was fixed to 59 wt% and mixing time of the third solution to 30 min. c) Electrical conductivities and anisotropy as a function of weight of the first solution and insulating to conducting phase ratios. In this particular case, DMSO content was fixed to 16 vol% and the mixing time of third solution to 30 min. White areas in the contour plots have conductivity less than 10^−5^ S m^−1^. Data were expressed in (b) and (c) as mean values (*n* = 5) for total of 90 compositions and 450 samples. Individual data points are shown in the Supporting Information. d) Stability of the multiphase conductor in ambient conditions and when placed under water. The underwater sample was stabilized in ambient conditions for >10^4^ s. e) Photographs of the freestanding multilayered conductor when elastically elongated to ≈2200% at strain rate of >1500% s^−1^. f) Stress–strain hysteresis of the conductor with (≈143% s^−1^ strain rate, gauge length of 7 mm). g) Conductivity change of a pristine conductor as a function of tensile strain. h) Temperature‐dependent conductivity of a pristine conductor plotted as *σ*(*T*)/*σ*(300 K) over temperature in Kelvin and as ln(1/*R*) over 1000/*T*,.

The evolution of morphology and structure was a result of phase separation that was controllable with several composition parameters and processing conditions. The multiphase conductors show a composition‐dependent anisotropic electrical conductivity (Figures S13 and S14, Supporting Information) that varied with the measurement direction (as illustrated in **Figure** **2**a). Linear *I*–*V* characteristics were observed when sweeping from −1 to 1 V (Figure S14, Supporting Information). The contour plots for the electrical conductivities were then obtained by measuring mean resistivity values (*n* = 5) and calculating the conductivities as a function of the composition and the processing condition for total of 90 compositions and 450 individual samples (Figure 2b,c). The discrete values are shown in Figures S15 and S16, Supporting Information. The direct current electrical conductivities parallel to stencil printing direction (*σ_x_*_‐axis_) increased from ≈10^−5^ to 10^−1^ S cm^−1^ when volume fraction of PEDOT‐rich nanofibrils increased from ≈0.1 to 0.6 vol%. This was due to increased average number of percolating connections with the assumption that no morphological and structural changes would simultaneously occur. The conductivity perpendicular to stencil printing direction (*σ_y_*_‐axis_) was lower than *σ_x_*_‐axis_ on the surface of the film that was highly conductive. We suppose that the stencil printing results in orientation of the PEDOT‐rich nanofibril network along the printing direction. It should be noted that the surface of the film that faced against the air‐interface was poorly electrically conductive (conductivity less than 10^−5^ S cm^−1^). The obtained conductivity was equal to or less than along the thickness of the film (i.e., *σ_z_*_‐axis_). The achieved levels of conductivity and electrical anisotropy are strong indicators of vertically phase‐separated organic conductor–elastomer blend with further consideration of the optical images and phase AFM images.

Even with low volume content of PEDOT‐rich nanofibrils, the achieved parallel conductivity (*σ_x_*_‐axis_) was superior to that of pristine PEDOT:PSS films (typically in a range of 0.1–1.0 S cm^−1^) after aging. The origin of higher conductivity was related to the conductivity enhancer that resulted in a morphological change for the PEDOT:PSS, and also due to existing vertical phase separation present in the multiphase conductor. Because numerous conductivity enhancer and strategies exist for enhancing both the flexibility and electrical conductivity of pristine PEDOT:PSS, we suppose there would be considerable possibilities to further improve the conductivity without compromising other properties in the multiphase conductor.^[^ ^46^ ^]^ However, the achieved levels of conductivity are already sufficient for many practical applications, such as for electroluminescent displays, bioelectrodes, electrodes or active piezoresistive sensing layers for soft sensors (important for wearable electronics).

**Figure 3:**
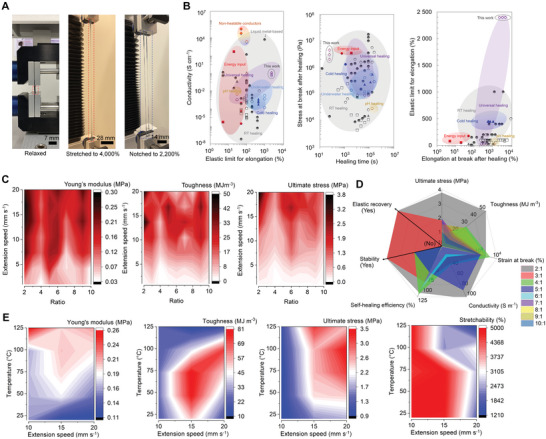
Tensile properties and performance comparison. a) Photographs of pristine and notched conductors under tensile strain (scale bars: 7, 28, and 14 mm; gauge lenght (*L*_0_) = 7 mm). b) Performance comparison to the state‐of‐the‐art self‐healing conductors and the best performing nonhealable conductors. Hydrogen bonds, metal–ligand interactions, and other mechanisms are denoted by circles, triangles, and squares, respectively. Filled data points present nontransparent conductors. Data point references can be found from the Supporting Information. c) Mean values plotted for Young's moduli (*E*; expressed as MPa), toughness (*U*_T_; expressed as MJ m^−3^), and ultimate stress (*σ*_max_; expressed as MPa) as a function of extension speed (equal to strain rate of 14.2% s^−1^–286% s^−1^ with *L*_0_ = 7 mm) and insulating to conducting phase ratio (2:1 to 10:1). d) Property comparison between pristine conductors. Data expressed as mean values (*n* = 3). e) Tensile properties plotted as a function of temperature (from 20 to 125 °C) and strain rate (≈14.2% s^−1^–286% s^−1^) with 2:1 ratio (*L*_0_ = 7 mm). Specific compositional information and discrete stress–strain curves for individual samples in (c–e) can be found in the Supporting Information. Data expressed as mean values (*n* = 3) for total of 63 and 25 measurement points in (c) and (d), respectively.

Strikingly, the PEDOT‐rich surface layer of the film had conductivities 10^2^–10^8^ orders of magnitude higher than with many of the recently developed state‐of‐the‐art solid‐state self‐healing conductors in their pristine conditions (shown in **Figure** **3**a; Table S1 and Figure S18, Supporting Information). However, this range excludes any liquid‐metal based conductors or conductive traces with out‐of‐the plane geometries due to obvious reasons. The limited conductivity of many of the previously reported self‐healing conductors is a considerable hindrance for practical application possibilities of these materials (other than for piezoresistive sensing layers). Although, particular design strategies have been able to considerably improve the otherwise limited electrical conductivity,^[^ ^47^, ^48^, ^49^ ^]^ the overall improvement has come at significant cost of multiple other important material level properties. This highlights the advantages of our approach where different material properties can be simultaneously optimized by using the heterogenous and cocontinuous morphology to achieve excellent toughness, elastic recovery, and faster and more efficient self‐healability (even in more challenging environmental conditions without any external energy or intervention).

### 2.6 Interdiffusion and Morphological Stability

Morphologically stable multiphase conductor compositions (Figure 2b,c) were found on the basis of electrical conductivity, for example, when a change of parallel conductivity (*σ_x_*_‐axis_) was measured as a function of time during aging (Figure S17, Supporting Information). The reason was that the achieved levels of conductivity related to the vertical phase separation which was also important for a long‐term morphological stability of the conductor. With a moderate to high insulating to conducting phase ratios, the conductors were trapped to metastable state after the polymerization. In this case, the metastability means that the multiphase conductors are not at their thermodynamic equilibrium when at relaxed state. We suppose the main reason is that the high molecular mobility of the surrounding chains present in the self‐healing multiphase conductors. Hence, over time, a thermodynamic relaxation occurs at the interphase regions which results in the interdiffusion in the multiphase conductor. The outcome of the phenomena is a homogenization of the multiphase conductor at the molecular and the macromolecular level which results in a change of material properties as a function of time. We suppose this phenomenon is alike to interdiffusion found in donor–acceptor blends.^[^ ^10^ ^]^

We suppose that the interdiffusion, in the multiphase conductors, were mainly dominated by the molecular weight of the soft phase. It is well‐known that the self‐diffusion coefficient (*D*_rep_) is highly dependent on the radius of gyration of a polymer chain (*R*_G_) and the longest relaxation time (*τ*_rep_ ∝ *M* ^3^) by *D*_rep_ ≈ (1/6)*R*_G_2/*τ*_rep_ ≈ *M* ^−2^.^[^ ^50^ ^]^ The reptation would be expected to be the mode of motion for the mobile polymer chains at the relaxed state.^[^ ^50^, ^51^ ^]^ Thus, the motion of the mobile polymer chains along their contour length could be restricted by a presence of large number of topological entanglements. We have introduced these into the multiphase conductor by increasing the molecular weight (*M*) of the soft phase above the critical entanglement molecular weight (*M >> M*_e_). Hence, we found that the interdiffusion took a quite long time for different multiphase conductors’ compositions at ambient conditions (at ≈20–23 °C, 20–30 RH%). The interdiffusion occurred over the course of ≈9 to 20 days within the transition region or above the region (Figure 2b,c). It should be pointed that the time taken to reach the thermodynamic equilibrium would be largely dependent on the ambient humidity levels and swelling (on the occasion when immersed to liquids) due to hydroscopic nature of PEDOT‐rich nanofibrils and presence of Si:O—B dative bonding.^[^ ^13^ ^]^ As shown, the interdiffusion results in a disappearance of vertical phase separation, thus loss of conductivity and electrical anisotropy.

The morphologically stable multiphase conductors maintain stable parallel conductivities (*σ_x_*_‐axis_) in both ambient conditions and under water even after several months of aging (Figure 2d). We assume that the water stable conductivity was a result of strong *π*–*π* interactions in the PEDOT‐rich nanofibrils and as negligible swelling of the conductor occurred with morphologically stable composition. The conductivity decreases until the swelling equilibrium has been achieved (≈4600 s) after which the conductivity starts to increase (≈12 000 s) until the value saturates above the initial value (≈13 500 s). The swelling behavior and anisotropic electrical properties of the swollen conductors require further investigation with consideration of the interdiffusion mechanisms and orientation of the individual films within the multilayered film geometry. The water stable electrical conductivity is crucial for any wearable or on‐skin electronics applications of the material, such as self‐adhesive bioelectrodes.

Below the transition region, the PEDOT‐rich nanofibrils (with an amphiphilic surfactant) may act in a similar manner as additives or nanoparticles commonly used to stabilize cocontinuous morphologies through improved cohesion.^[^ ^47^, ^48^ ^]^ As shown, the multiphase conductors can be trapped into a thermodynamic equilbria by fine tuning several compositional parameters and processing conditions that have influence on the morphology and the structure formation. Specifically, at low insulating to conducting phase ratios heterogenous cocontinuous multiphase conductors can become morphologically stable with a formation of well‐defined vertical phase separation (Figures S5–S8, Supporting Information). Hence, we suppose that a high enough content of organic conductor and specifically a high molecular weight are key requirements in order to achieve long‐term morphological and structural stability. Similarly as previously discussed,^[^ ^10^ ^]^ we suppose the interactions between the interphase regions in the multicomponent blend must become so unfavorable that the motion of mobile polymer chains becomes highly restricted, and the morphology freezes in a place when at relaxed state. Although structural degradation would be prevented on both shorter and longer time scales in the conductor, this would not result in inability to switch between energetically higher and lower states. The reason is that the stimuli‐responsiveness is still maintained by the presence of supramolecular interactions acting as reversible dynamic cross‐links.

Interestingly, the multilayered films, stacked by utilizing self‐bonding of single layers, showed an increased morphological instability during the aging. Thus, overall thickness of the films and the number of layers becomes important considerations for the thermodynamic stability, molecular mobility, and swelling behavior of multiphase material. It should be noted that the morphological instability issues may even exist with single‐layered films when the thickness considerably increases. One of the reasons could be that the increased film thickness results in a high degree of domain coarsening and reduces the degree of interpenetration in the network (that both result in instability). Hence, morphologically stable multiphase conductors can be obtainable only with relative thin films. Because interdiffusion of phases may take even longer than several months of aging, further studies are required to fully understand the composition‐dependent interdiffusion mechanism.

### 2.7 Electromechanical Properties and Temperature Dependency

The multiphase conductors that have large phase‐separated domain sizes show extraordinary levels of elasticity not previously achievable with any other self‐healing conductor. The stretchable conductor was capable of recovering from extremely large deformations due to entropy‐driven elasticity (Figure 2e–g). The ultraelastic nature of the conductor (Movie S1, Supporting Information) was reliant on the heterogeneity and microphase‐separated interfacial regions that are capable of proving highly stable junction points under strain (as illustrated in Figure 1c). This restricts the irreversible flow of the self‐healing conductor under extremely large tensile strains.^[^ ^13^ ^]^ Surprisingly, even after the heterogenous cocontinuous multiphase conductors fractured at tensile strains (*ε*) of ≈2000–3000% only a small residual strain existed with a fast strain rate (less than 100%). At lower strain‐rates, a nonaffine macroscopic deformation may exist in the conductor and result in irreversible deformation at extremely large tensile strains.

A relatively small stress–strain hysteresis exists as the conductor stabilizes itself after ≈5 consecutive strain–release cycles in the range of *ε* ≈ 190–1100% (Figure 2f). The hysteresis was considerably smaller than for the electrically nonconductive self‐healing elastomer.^[^ ^13^ ^]^ Hence, the fatigue resistance of the conductor would be superior upon dynamic loadings which is highly advantageous for soft electronics. When a parallel conductivity change was measured under tensile strain (Figure 2g), the ratio of strained conductivity to nonstrained conductivity (expressed as *σ_*ε*_*/*σ*_0_) was in the range of 10^0^–10^−1^ at *ε* = 0–200%. The piezoresistive gauge factor under tensile strain increases from ≈4.02 to ≈360.10 as the extensibility exceeded 200% strain region. The reversible dislocation of PEDOT‐rich nanofibril network results in an increase of the *σ_*ε*_*/*σ*_0_ with the tensile strain. It should be pointed out that even the strained conductivity was considerable better than the nonstrained conductivity of many other self‐healing conductors (as shown in Figure 3b) and the entropic elasticity allows recoverable conductivity even after stretching over the region of high conductivity. The shape recovery and phase rearrangements were possible even after large deformations (up to *ε* ≈ 2000%) upon release of a strain. Although, other types of self‐healing conductors have been found highly stretchable, the elastic region was severely limited due lack of entropic elasticity. Hence, stretching over the elastic region would typically result in irreversible deformation and permanent loss of conductivity. The entropic elasticity makes the multiphase conductor extremely resilient for deformations that may even exceed its functional working range (dependent on tolerable change of conductance) when used as an electrical wire, contact or electrode in soft electronics. Depending on the application, the conductive change under strain can be further reduced by using strain‐insensitive geometrical designs. The ultraelastic nature of conductor becomes particularly advantageous for piezoresistive strain sensors, or when utilized in soft actuators that are capable of stretching multiple times of their original length.

Temperature dependent conductivity behavior of disordered conducting polymers implies that a charge transport is dominated by the interchain hopping conducting mechanisms. To evaluate the charge transport behavior of the multiphase conductor, temperature dependent parallel conductivity was plotted as *σ*(*T*)/*σ*(300 K) over the temperature (Figure 2h). The temperature‐activated conduction in the multiphase conductor follows the Arrhenius law (due to Gaussian disorder) while the positive slope was an indication of the thermally assisted hopping conduction between the localized sites. Upon initial heating cycle, we found that parallel conductivity (at the highly conductive surface) considerably increased around the cross‐linking temperature of the multiphase conductor (343.15 K). Remarkably, upon cooling down and then heating the second time, the linear temperature resistance coefficient (defined from *σ_x_*_‐axis_) decreased from a maximum value of ≈3.02% to 0.63% K^−1^. After resting for a day in ambient condition, the conductivity recovered to the base value before the temperature sweeping and the anisotropy (defined as *σ_x_*_‐axis_/*σ_y_*_‐axis_) was preserved. Then, similar temperature dependent conductivity change and hysteresis loop were observed upon similar heating and cooling cycles (as shown in Figure 2f–h). We suppose the elevation of PEDOT‐rich islands increased upon the heating (close T the cross‐linking temperature) resulting from the conformational change in the conductor (Figures S9 and S19, Supporting Information). However, to fully understand the temperature‐dependent electrical properties and structural changes (upon the temperature sweeping) would require further investigations with more detailed analysis.

### 2.8 Tensile Properties as a Function of Strain Rate and Temperature

To fully understand the complex tensile property dependency on the composition and structure, electrically nonconductive elastomer films were first prepared with fixed thickness (Figure S20, Supporting Information). We then compared tensile properties of self‐bonded multilayered films to single‐layered films. The thickness normalized tensile properties and self‐healability improved when using multilayered film geometries. For instance, a toughness increased from 30 to 60 MJ m^−3^ as the extensibility increased in expense of stiffness. The shape recovery and time required for self‐healing, after elongated to break, decreased from ≈7200 to 1250 s. This suggests a higher molecular mobility in the multilayered films by simply laminating two similar films together. Thus, we have adopted the multilayered films structures for the remainder of this work (Figure 3a–e). Another important aspect was that the thickness of individual films cannot be considerably increased without influencing the morphology and structure of the multiphase conductor.

The Young's moduli (*E*) for multiphase conductors were in the range of ≈0.1 to 0.4 MPa at small strains (*ε* < 50) with a slow strain rate of ≈14.2% s^−1^ (Figure 3c–e). This was close to the electrically nonconductive elastomer reported in our previous work.^[^ ^13^ ^]^ Thus, introducing a small volume fraction of PEDOT‐rich nanofibrils into the multicomponent blend does not result in an increase of stiffness. At low strain region, the stiffness was independent whether the elastomer was in dehydrated, hydrated, or swollen state (when exposed to humid conditions or swollen in liquids). Also, the measured values for *E* were found to be close approximate of the theoretically calculated values derived from the equivalent Takayanagi model designed for multicomponent systems (Figure S21 and Table S3, Supporting Information). Hence, stiffness was largely dependent on the intrinsic tensile properties of the electrically nonconductive self‐healing elastomer.

The complex composition‐dependent viscoelastic behavior was a result of heterogeneity, coexistence of multiple phases, and their interphase regions with distinct stress–strain relaxation characteristics.^[^ ^52^, ^53^ ^]^ Thus, the tensile properties are highly dependent on the strain‐rate and temperature with all different compositions. The contour plots of the tensile properties (Figure 3c–e) were obtained by plotting the mean values (*n* = 3) for total of 63 and 25 individual measurements points, respectively. Linear stress–strain characteristics were observed for the multiphase conductor only when the strain rate considerably increased. It is well known that only some of the polymer chains carry most of the tensile loading. Hence, the macroscopic deformation in the multiphase conductor (independent of the equilibrium state) was controlled by the PEDOT‐rich nanofibrils that carry most of the tensile loading and can act as reinforcing filler. This may occur with the consideration of improved contact area through a denser nanofibril network and due to interphase mixing as the surface tensions reduce and the interfacial adhesion increases through the amphiphilic surfactant. The nonmonotonous change of tensile properties with the composition relates to the simultaneously occurring morphological and structural changes in the multiphase conductor. The optimal multiphase conductors in terms of tensile properties are then found on the basis of electrical conductivities (Figure 3d; Figure S22, Supporting Information) that reflects the cocontinuous structure important to achieve excellent overall performance for the self‐healing conductor.

The self‐induced stiffening with the expense of extensibility as a function of strain rate and/or temperature can be explained by the molecular interlocking mechanism present in the soft phase.^[^ ^13^ ^]^ Hence, some multiphase conductors could effectively dissipate the strain energy in a broad strain‐rate and temperature ranges without network fracture (independent of whether the elastomer network was in dehydrated or hydrated state). The extensibility of the multiphase conductor could exceed 5000% strain which was over 1000 times better than with pristine PEDOT:PSS films having a fracture strain of ≈5%. A record‐high toughness was achieved (Figure 3b) that exceeded 80 MJ m^−3^. This level of toughness was achievable as both the strain‐rate and the temperature increased with conductors that were not swollen in liquids (Figure 3d,e; Figures S23–S25, Supporting Information).

The reversible softening of the multiphase conductor (e.g., when swollen in high relative permittivity liquid) results in a decrease of toughness when the surrounding elastomeric matrix (around the PEDOT‐rich nanofibrils) becomes softer as all polymer chains participate in the swelling. In comparison, the extensibility of the electrically nonconductive elastomers was severely restricted due the absence of PEDOT‐rich nanofibrils (Figure S20b,c, Supporting Information). The elastomers fractured at significant lower strain (≈300–1000%) than the multiphase conductors when strain‐rate and/or temperature was increased. The same molecular interlocking mechanisms made the heterogenous cocontinuous both stronger and tougher with the *π*‐conjugated polymer present.

The multiphase conductor having vertically phase‐separated interpenetrated network was flaw insensitive under tensile testing (Figures S26 and S27, Supporting Information). The flaw insensitivity was absent in the electrically nonconductive elastomer reported in our previous work.^[^ ^13^ ^]^ Remarkably, the multiphase conductor could be stretched to a similar degree regardless of the size and shape of the crack. The flaw insensitive behavior is not typical for elastomers, or stretchable conductors, especially as larger cracks easily propagate in single elastomeric networks (Figure S26, Supporting Information). Based on our previous work, we suppose that the hard phase was capable of rather homogenously carrying the tensile loading. Upon rupture of hard phase, the tensile loading would be then transferred to a soft phase that acts as a sacrificial network. Hence, cracks would be able to easily propagate in the self‐healing elastomer once the hard strands are nearly fully extended from their coiled conformation (around 150% tensile strain).^[^ ^13^ ^]^ The contour plots and discrete tensile stress–strain curves (Figure 3c,d; Figures S23–S25, Supporting Information) show that the PEDOT‐rich nanofibril network carried most of the tensile loading. Hence, the crack growth and propagation were prevented as any stress concentrations would be avoided trough the efficient strain energy dissipation by both the sacrificial network and the rupture of the hard strands.

### 2.9 Universally Autonomous Self‐Healing

It has been challenging to achieve an excellent electromechanical performance and self‐healability in a stretchable conductor while also maintaining the said functionalities in more extreme environments (Figure 3b). The environmental resistance of self‐healable functional materials becomes crucial, for example, for wearable electronic devices as exposure to water, sweat, and high‐humid conditions cannot be avoided. Autonomous self‐healability has been difficult to achieve without the expense of mechanical and electrical properties in low or high pressures and temperatures, saturated salt concentrations, highly acidic or alkaline conditions, or combinations of previously mentioned. Each of these environments may present a considerable technical challenge to initiate the self‐healing at the material level. However, we show that the combined chemico‐physical self‐healing would be a potential route to achieve autonomous self‐healability in all of the above‐mentioned conditions with a phase‐separated interpenetrated network and use of supramolecular elastomer.

**Figure 4:**
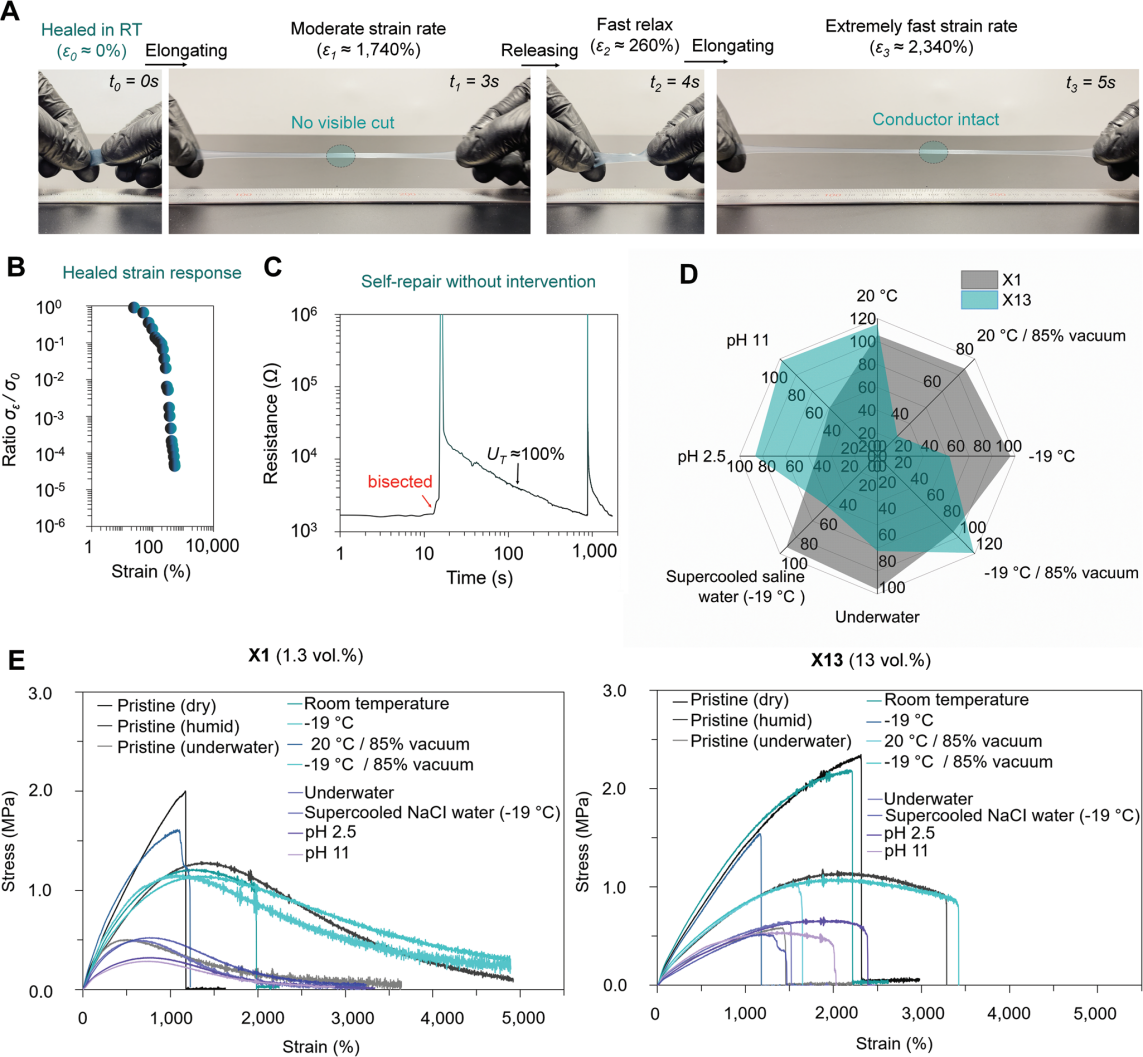
Self‐healing properties. a) Photographs of self‐healed, freestanding multilayered conductor under manual stretching. b) Change of electrical conductivity under tensile strain for the self‐healed conductor. c) Time‐dependent resistance change of a conductor when self‐healing without intervention in ambient conditions. The sample was bisected two times with a razor. During the measurement, *U*_T_ = 100% indicates a point of time when toughness has been fully recovered. d) Radar plot comparison of self‐healing characteristics for conductors that have different amount of surfactant. Data expressed as mean values (*n* = 3). e) Tensile stress–strain curves for pristine and healed conductors in different conditions, including room temperature (20 °C), cold (−19 °C), and/or in vacuum, saline, acidic, and alkaline conditions (strain rate 142.8% s^−1^).

The ultratough and resilient multiphase conductors exhibited ultrafast and autonomous self‐healing. The conductors, with optimized composition, fully recovered the pristine tensile properties in a less than ≈120 s after the initial contact of the cut‐surfaces. The self‐healability was found to be highly dependent on multiple factors, such as composition of the siloxane‐based elastomer,^[^ ^13^ ^]^ heterogeneity, formation of a PEDOT‐rich nanofibrils, and vertical phase separation (Figures S22–S25 and Table S3, Supporting Information). For the multiphase conductor compositions that had excellent self‐healability, the mechanical cut was not clearly visible, or it was only visible under tensile strain (**Figure** **4**a). The self‐healing can be repeated multiple times with similar efficiency without loss of properties or cut‐surface aging effects. In the dynamic state (when mechanically damaged), band intensities change in the multiphase conductors were similar to the electrically nonconductive elastomer (Figure S3c, Supporting Information). The band intensities change at the wavenumbers of 700 and 3290 cm^−1^ indicate that the Si:O—B dative bonds and the boron‐containing functional groups are reforming bonds with the siloxane‐backbone present in the conductor.^[^ ^13^ ^]^ However, a visible spectral features changes occurred in wavenumbers of 1300–1600 and 1800–2500 cm^−1^. This could be due to the reversible structural changes occurring in the conductor, upon phase rearrangements, and self‐healing of the PEDOT‐rich nanofibril network. However, origin of such spectral features is yet unknown and requires a further investigation.

As the self‐healability of the conductor was reliant on both the chemical interactions and physical elastomeric network properties, there are multiple factors contributing to the ability to repair mechanical damages. The stages of the self‐repair are alike to the crack healing. Rather than simply regaining the electromechanical properties through a time‐dependent diffusion of polymer chain segments (reliant on the radius of gyration of a polymer chain ascribed by the de‐Gennes reptation model),^[^ ^37^ ^]^ there exist also intermolecular interactions. The dipole–dipole interactions (O—H and O—B bonds) result in bond dissociation and dislocation which are considered to be energetically and spatially favorable for the phase rearrangements.^[^ ^38^, ^54^ ^]^ The high flexibility of multimodal polymers chains (present in the conductor) allows to achieve large number of chain configurations important for the fast self‐healing by increasing the change of Δ*G*. At the same time, the heterogeneity (in terms of localized variations of the glass transition temperatures) results in high free volume near the damaged sites which also contributes to the speed of self‐healing through increased diffusivity.

The presence of dual‐rich regions of the PEDOT‐rich domains and the hard phase‐rich domains significantly increased the molecular mobility of the multicomponent material system (Figure S25, Supporting Information). The anisotropic electrical properties (Figure 2b,c) directly reveal the compositions with long‐term morphological stability and excellent combination of electrical, mechanical, and self‐healing properties. The multiphase conductors, with optimal composition (Figure 3d **;** Table S3, Supporting Information), show ultrafast self‐repair with a cut‐surface alignment. The achieved autonomous self‐healing was 10^2^–10^4^ orders of magnitude faster than with any of the currently existing self‐healing conductors having solid state properties (*σ*_max_ ≥1 MPa after self‐healing). Remarkably, the autonomous self‐healing was also over 10^2^ orders of magnitude faster than in our previous work^[^ ^13^ ^]^ (decreased from ≈7200 to 120 s) while the tensile properties simultaneously improved. We suppose that the extraordinary self‐healing characteristics relate to the controllable heterogeneity and vertically phase‐separated multilayered structures. The available high free volume and the existing compositional gradients (through the existing polymer domains and interphase regions) improve the segmental mobility of macromolecules and decrease the time required for the phase rearrangements through hydrophobic and hydrophilic interactions.

**Figure 5:**
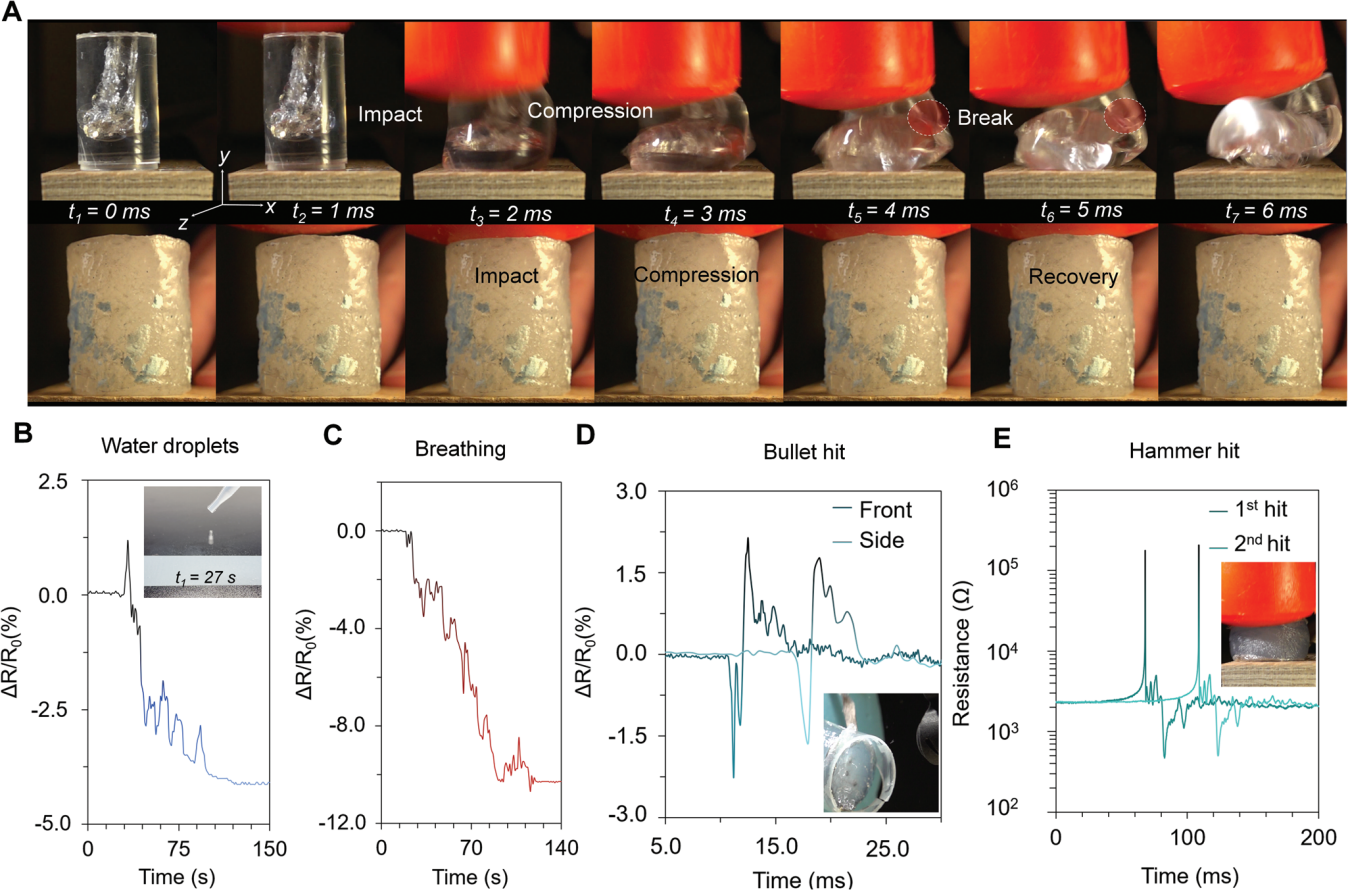
Damage tolerance and piezoresistive sensing. a) Photographs of cylinder‐shaped polydimethylsiloxane and self‐healing elastomer (without conductive coatings) after shot repeatably and then striking with a hammer. b–d) Relative resistance change (Δ*R*/*R*_0_) of the multiphase conductor coatings with water droplets, when breathing to a surface of the film, and upon the impact of a bullet from the front and side of the cylinder through the conductive coating. e) The change of Δ*R*/*R*_0_ upon impacts when striking with the hammer.

The restoration of anisotropic conductivity was highly dependent on the of PEDOT‐rich nanofibril network due to existing vertical phase separation. Hence, the full conductivity recovery (≈900 s) took a significantly longer time (**Figure** **5**b,c) than the ultrafast recovery of toughness (60 to 120 s) after a similar mechanical damage. This was because the wound closure propagates from the bottom of a scratch driven by both the interfacial and entropic energy as the radius of curvature in the location of cut further increases.^[^ ^13^, ^54^ ^]^ Hence, significant portions of the conductivity become recovered only after the scratch is completely healed. However, as shown, the complete disappearance of the scratch was not required to achieve full recovery of tensile properties (with cocontinuous multiphase conductor). As shown, the healed conductor fully recovers *σ_*ε*_*/*σ*_0_ under tensile strain only after the wound closure was fully complete. This indicates that the PEDOT‐rich nanofibril network recovers same level of mechanical integrity that was present in the pristine sample (Figure 5c). This means that the PEDOT‐rich nanofibril network was also capable of autonomously self‐healing likewise as the elastomeric matrix. It should be further pointed out that a large surface area self‐healing without any intervention was significantly slower than for a single cut with cut‐surface alignment (Movie S2, Supporting Information). The perforation made to material does not fill by the flow of the material which means the material was capable of recovering its original shape.

The self‐healing of tensile properties was considerable faster than for electrical conductivity. This was inverse to that of isotropic self‐healing conductors with surface conductivity similar to the bulk conductivity. The inverse self‐repair has considerable major advantages for applications related to soft electronics and robots. More importantly, the flaw insensitive elastomeric network can withstand large deformations after a short healing period due to rapid recovery of the tensile properties. Both would prevent the healed conductor rupture upon static or dynamic loading conditions, while the actual damaged location would be still distinguishable through increased resistivity. Because conventional self‐healing conductors that have electrical percolation networks lack a well‐defined vertical phase separation, the conductivity may either fully or partially restore with the initial physical contact of the cut‐surfaces. However, the actual recovery of tensile properties and mechanical integrity of the percolated network under tensile strain would be still limited and the recovery may take a very long time (multiple hours, or even days, to complete at optimal conditions; Figure 3b). Because self‐healing conductors often lack fatigue resistance and flaw insensitivity, they would remain extremely long time in a mechanically nonfunctional state. These vulnerable elastomeric networks may rather easily rupture with static and dynamic loading conditions before fully healed.

The heterogenous cocontinuous multiphase conductors achieved excellent and environmentally resistant autonomous self‐healing characteristics in universal conditions (Figure 5d,e; Table S4, Supporting Information). Due to reversible softening of the multiphase conductors and the elastomers (when exposed to humid conditions or immersed to liquids),^[^ ^13^ ^]^ the self‐healing efficiencies were calculated in comparison to pristine conductors that were either dehydrated (kept at 70 °C), hydrated (kept at ambient conditions), and swollen in the water (the immersion time was fixed to 60 s). The tested conditions include low pressure (at ≈15.2 kPa, and 20 °C or −19 °C), low temperature (−19 °C), underwater (20 °C), in supercooled saline water (26.3 wt% of NaCl at −19 °C), acidic (pH = 2.5 at 20 °C), and alkaline (pH 11 at 20 °C) conditions with 32–120% recovery of *U*_T_ in 60–120 s (Figure 5e) with cut‐surface alignment. More importantly, all other tensile properties of the conductor also fully recover. Although, many PEDOT:PSS‐based self‐healing conductors have been previously reported,^[^ ^48^, ^49^, ^55^, ^56^, ^57^ ^]^ this work was the first to achieve universally autonomous self‐healing with PEDOT:PSS. The excellent ultrafast, autonomous, and environmentally resistant self‐healing of the multiphase conductor would be use for wearable devices and on‐skin electronics. Interestingly, the multiphase conductor, to the best of our knowledge, was the first electrically conductive material capable of self‐healing in the low pressures (i.e., in a vacuum) without external energy or intervention. Due to extraordinary tensile properties and environmentally resistant self‐healability, the multiphase conductor may find many interesting applications which are not possible with other type of self‐healing conductors.

### 2.10 Damage Tolerance and Piezoresistive Sensing Functionalities

Furthermore, we demonstrated damage tolerance and piezoresistive sensing functionalities (Figure 5a–e) of the self‐healing elastomer and multiphase conductor coatings. We first made cylinder‐shaped sample of the electrically nonconductive elastomer (see the Supporting Information) that was shot repeatably. The bullets easily passed through the cylinder without any visible damage to the sample (other than gunpowder at the bullet entry points). Upon initial impact, when striking with a hammer, the cylinder became extremely stiff due to shear stiffening effect. Hence, the sample compressed only by ≈3.3% (Figure 5a; Movie S3, Supporting Information). The relative compression further increased up to ≈49–52% upon the second and the third strike due to elastic hysteresis (Movie S3, Supporting Information). We were unable to break or induce any permanent deformation to cylinder‐shaped sample even after countless of strikes at full force (after first shooting it repetitively). The blue parts in the photographs (Figure 5a) are pieces of plastics adhered to the sample after the cylinder‐shaped sample was removed from the sample holder for the impact testing. In comparison, a pristine cylinder made of Sylgard 184 poly(dimethylsiloxane) (PDMS) easily fractured upon the impact when compressing by ≈55% of its initial height (Figure 5a). The fracture occurred only after three consecutive shots and with the second strike with a hammer (Movies S4 and S5 and Figure S28, Supporting Information).

To further demonstrate the excellent elasticity and to show the piezoresistive force sensitivity, we then transferred a multiphase conductor film from polyethylene terephthalate (PET) film to a cylinder‐shaped electrically insulative self‐healing elastomer (please see the Supporting Information). We were then able to record in a real‐time the impact of bullets and hammer strikes with the piezoresistive multiphase conductor coating. The bullets travelling at >300 m s^−1^ passed through cylinder in less than 0.3 ms. The impact could be easily measured in a real‐time due to extremely fast response time and efficient kinetic energy transfer from the cylinder to the conductive coating (Figure 5d). The relative resistance change (Δ*R*/*R*_0_) was larger when the coated cylinder was shot along the length of the cylinder rather than from the side. This can be explained by transverse expansion of the cylinder along its cross‐sectional area as the kinetic energy of the bullet is transferred to the surrounding self‐healing elastomer matrix. The conductive coating was undamaged by the bullets and the durability was tested under extreme impacts (Figure 5e). The shot cylinder was repeatably hit with a hammer. After repeatably striking the cylinder, we were still able to receive electrical signal without any signal distortion. Also, we found no physical damage in the coating or cylinder after the testing was complete (Figure S28, Supporting Information).

The excellent potential of the multiphase conductor for smart skins and soft sensor applications was further demonstrated with piezoresistive humidity sensing. The highly hydroscopic nature of PEDOT‐rich nanofibrils and water sensitivity of Si:O—B dative bonding results in high sensitivity to humidity and presence of water molecules. Ultrasensitive nature of the multiphase conductor coating on PET was demonstrated by measuring impacts of tiny water droplets and air flow when breathing to the surface of a film (Figure 5c,d). This was when the highly conductive surface of the multiphase conductor film (in a nonswollen equilibrium state) faced against the air interface. The impact force of a water droplet was approximated to be ≈28.73 Pa (see the Supporting Information). Hence, the films have extremely low limit of detection for mechanical force. Thus, the multiphase conductors could be applied in future for self‐adherable on‐skin electrodes or sensors capable of measuring, for instance, muscle contraction or physiological signals. The impact of the droplets results in increase of Δ*R*/*R*_0_ by ≈1.1% corresponding to estimated force sensitivity of more than 0.38 kPa^−1^ with simplistic films geometries. The piezoresistive force sensitivity could be considerable increased, for example, by further tuning the composition for improving the mechanical compliance. The spreading of a water on the surface of film is seen as a surge of Δ*R*/*R*_0_ after the initial impact (*t*_1_ = 27 s) with the break of surface tension in the water droplet. We suppose that the absorbed water molecules result in increase of conductivity over time due to reversible localized conformational changes occurring in the PEDOT‐rich nanofibril network. As a result, Δ*R*/*R*_0_ decreases over time before the piezoresistive response stabilizes in ≈20 s after the last impact of water droplets.

The change of Δ*R*/*R*_0_ (≈1.5–2.0%) under the breathing was significantly larger than upon the impact of a water droplet (when at a nonswollen equilibrium state). Change in the magnitude of the air flow was clearly visible from the Δ*R*/*R*_0_. The piezoresistive response recovers only after a few seconds to the base noise level when the air flow stops (Figure 5c). The decrease of Δ*R*/*R*_0_ over time was due to the water vapor from the breathing air as the water molecules penetrate the surface of the film (resulting in a conformation change). In both above‐mentioned cases, the conductivity recovers to the initial value as the water molecules evaporate from the film. However, this can take a considerable time without any intervention in the ambient conditions.

## 3 Conclusion

Herein, we reported a novel flaw insensitive multiphase conductor with cocontinuous morphology, heterogeneity, and macroscale phase separation to achieve ultrafast and efficient autonomous self‐healability in universal conditions. The multiphase strategy, with vertically phase‐separated interpenetrated network, allowed to synergistically achieve an unprecedented combination of electrical, mechanical, and self‐healing properties in a soft conductor. We anticipate that, with a future work, the material design concept is applicable to many other organic conductor–elastomer blends to enable spontaneous self‐healing with minimal external intervention. We expected that the solution‐processable multiphase conductor may find many potential application areas, for example, as transparent touch‐sensitive surfaces, soft sensor/actuator components, self‐adhesive bioelectrodes, or electrodes in electroluminescent displays, electronic or photonic smart skins with all built‐in material level self‐healing.

## 4 Experimental Section

### Materials

Hydroxyl terminated poly(dimethylsiloxane) (PDMS‐OH) with kinematic viscosity of 18 000–22 000 cSt, dimethyl sulfoxide (DMSO; 41640‐M. ACS reagent ≥ 99.9%), and Triton X‐100 (laboratory grade) were purchased from Sigma‐Aldrich. Poly(3,4‐ethylenedioxythiophene):poly(styrenesulfonate) aqueous solution (PH1000) was purchased from Ossila Ltd. Boron oxide nanoparticles (B_2_O_3_ NPs) (*d*_50_ = 80 nm) were purchased from SkySpring Nanomaterials Inc. Dimethylvinyl‐terminated dimethylsiloxane‐based elastomer (hard phase) was purchased from Sil‐mid Limited. The silver‐plated nylon fabric (P130) was purchased from Shieldex. All materials were used as received.

### Preparation of Multiphase Conductors

The first solution consists of dispersion of aqueous PH1000 and DMSO. The added DMSO content to the defined amount of PH1000 was varied from 0 to 48 vol%. The first solution was mechanically mixed by a magnetic stirrer bar to concentrate the solution by evaporating water from the aqueous PH1000. The weight of the first solution needs to be precisely controlled from ≈82 to ≈36 wt% (i.e., reducing the weight by ≈18–64%). It should be emphasized that direct blending of all components without any weight reduction was not feasible. The mixing time needs to be adjusted depending on the ambient conditions, rotation speed of the magnetic stirrer bar, and by the amount of PH1000 by continuously measuring the weight of the solution. For a typical experiment ≈2.654 g of mixture of PH1000/DMSO was magnetically stirred for ≈3.5 h with a rotation speed of ≈200 rpm to reduce the weight by ≈40.5%. For example, larger amount of PH1000 would require a longer mixing time, and vice versa for smaller amount. The second solution consisting of a bimodal siloxane‐based self‐healing elastomer (having a long and short polymer chain segments) was prepared as previously described.^[^ ^13^ ^]^ ≈0.82 wt% of B_2_O_3_ NPs (in relation to the bimodal self‐healing elastomer components) was mixed in a mortar with PDMS‐OH and polymer base of the hard phase when the first solution was nearly ready. After that the cross‐linking component of the hard phase was added to the second solution. The mechanical mixing of the first solution (with desired weight) was then stopped for a short period of time. The second solution (with cross‐linker) was then immediately added to the first solution (having the desired weight). Insulating to conducting phase ratios were defined as ratios of the second solution (with cross‐linker) and the first solution (without weigh reduction and with a defined amount of Triton X‐100). The ratios were varied in the range of 10:1 to 2:1. A defined amount of Triton X‐100 (0–15 wt% in relation to the amount of PH1000 at the start) was then immediately added to a mixture of the first and the second solution to homogenously disperse the components when using aqueous solution of PEDOT:PSS. The final solution was then referred as the third solution. The mechanical mixing of third solution was then continued from 10 to 60 min before it was ready for film preparation. The third solution was stencil printed onto a substrate. It should be pointed out, that the viscosity of the third solution increases over time during mechanical mixing and a minor composition‐dependent weight reduction still occurs. The cross‐linking of the film was then carried out at elevated temperatures (at 70 °C for 24 h). It should be noted that the thickness of the deposited films needs to be controlled in order to achieve a desirable morphology and structure in the conductor. In this work, the thicknesses of individual multiphase conductor films were ≈250 µm when prepared on the release film.

### Characterization

Fourier‐transform infrared spectroscopies were performed with Thermo Scientific Nicolet iS5 with D7 Diamond ATR and Nicolet iN10 from wavenumbers 4000 to 400 cm^−1^. Optical transmission measurements were taken with UV–Vis–NIR Varian Cary 500 spectrophotometer from 400 to 800 nm wavelength with sample thicknesses of ≈25 µm. Surface topology was assessed with Bruker MultiMode 8 Atomic Force Microscopy in tapping mode with HQ:DPE‐XSC11 type C cantilever with 7 N m^−1^ force constant and 155 kHz resonant frequency. Optical images and imaging were taken with an Olympus BX51 microscope with U‐POTP3 polarizer. Thermogravimetric analysis and differential scanning calorimetry were performed with NETZSCH STA 449F3 Jupiter from 30 to 550 °C in an oxygen atmosphere with a heating rate of 10 °C min^−1^. Measurements were calibrated with references. In all other cases, the characterizations were done with multilayered multiphase conductors when thicknesses were fixed to ≈1.0 mm.

### Tensile and Self‐Healing Properties

Tensile properties were measured with Stable Microsystems Texture Analyzer TA750 with 50N‐load cell using pneumatic clamps. The multilayered multiphase conductors had rectangular geometries with dimensions of ≈25–35 mm in length, 10–15 mm in width, and 1 mm in thickness. The multilayered films were made by self‐bonding four individual films together. The thicknesses of individual films were ≈250 um. The thicknesses of electrically nonconductive elastomer films were ≈1.0 and ≈2.0 mm. These multilayered multiphase conductor films were used for tensile testing. This was done in order to achieve a large enough thickness for easy handling of the samples while still maintaining desirable morphology and structure in the films. The tensile property measurements were done in ambient conditions (≈20–23 °C and 20–30 RH%) with extension speed of 1.0–20.0 mm s^−1^. This corresponds to a strain‐rate of ≈14.2–285.7% s^−1^ with fixed gauge lengh (*L*_0_) between the clamps (*L*_0_ = 7 mm). Due to extremely large deformations, true stress was calculated from the nominal stress with an assumption that elastomers were isotropic and incompressible by their volume. Unless otherwise stated, the tensile testing was performed after cross‐linking and/or after assembly of the freestanding multilayered films.

For self‐healing tests, the samples (with the above‐mentioned dimensions) were bisected with a razor perpendicular to the direction of uniaxial elongation. Unless otherwise indicated, the dissociation areas were 100% of the cross‐sectional area of the sample. The cut‐surfaces were manually aligned within ≈10 s (in all conditions tested). For low pressures, the samples were first damaged in ambient conditions and then immediately placed in a vacuum chamber that had an absolute air pressure of ≈15.2 kPa. This was ≈15% of the atmospheric pressure (≈101.3 kPa). To test the surface aging effects, bisected specimens were kept apart for 24 h at ambient conditions before cut‐surface alignment. The self‐healing efficiency was defined as a proportion of toughness recovered in relation to pristine samples (in the same condition). The tensile properties were measured from pristine samples that were either dehydrated (kept at 70 °C), hydrated (kept in the ambient conditions), or slightly swollen in water (the immersion time was fixed to 60 s).

### Electrical Properties

Anisotropic direct current electrical conductivities (*σ*_dc_) were calculated as a reciprocal of the resistivity (*σ*_dc_ = *ρ* ^−1^ = *R*_s_^−1^ *t* ^−1^, where *R*_s_ was sheet resistivity and *t* was film thickness). The thicknesses of the individual films were fixed to ≈250 µm. Large film samples were then cut accordingly for the electrical measurements (please see the Supporting Information for the illustration). The current–voltage (*I*–*V*) characteristics and resistance values were obtained with Keithley 2400 SMU and Keysight Electrometer B2987A, respectively. The *I*–*V* characteristics were measured by sweeping from −1 to 1 V. The measurements were done in ambient conditions (≈20–23 °C and 20–30 RH%). The anisotropy was defined as a ratio of vertical (*σ_x_*_‐axis_) to parallel conductivity (*σ_y_*_‐axis_). The *σ_x_*_‐axis_ value was measured for both sides of the film. Through conductivity (*σ_z_*_‐axis_) was defined along thickness of a sample from the top surface to the bottom surface of the film. To avoid issues with a high contact resistance, stretchable and conductive silver‐plated nylon fabrics were used to connect the probes to the conductive films. Average thickness along the length of a film was obtained to calculate the conductivity. To avoid undesirable effects by the environmental fluctuations or issues with morphology instability of the films, the resistivities were obtained directly after cross‐linking was complete and cooling at room temperature. The electrical conductivity under tensile strains were measured with the Stable Microsystem Texture Analyzer model TA750 by using laboratory made clamps with electrical wiring connected to the resistance meter.

### Statistical Analysis

Continuous variables were expressed as mean ± SD (*n =* 5; Figure 2b,c; Figures S15 and S16, Supporting Information) and mean (*n* = 3; Figures 3d and 4d; Figure S22, and Tables S3 and S4, Supporting Information). The data were not preprocessed, statistical tests or any software's were not used for the analysis of significance.

## Conflict of Interest

J.T., M.N., J.H., J.J., and H.J. have issued patent applications (EP21172728.4, EP21172721.9, EP22162435.6, and EP22162437.2) related to processes, materials, and devices described in this article.

## Supporting information

Supporting Information

Supplemental Movie 1

Supplemental Movie 2

Supplemental Movie 3

Supplemental Movie 4

Supplemental Movie 5

## Data Availability

The data that support the findings of this study are available in the supplementary material of this article.
