# Supplementary material for: Ultraelastic and High‐Conductivity Multiphase Conductor with Universally Autonomous Self‐Healing
Source: Adv Sci (Weinh). 2022 Nov 9;9(36):2205485. doi: 10.1002/advs.202205485 (PMC9798996; doi:10.1002/advs.202205485)
Supplement: Supplementary file 1 — Supporting Information [file ADVS-9-2205485-s002.pdf]

ADVANCED SCIENCE
Open Access

Supporting Information

for *Adv. Sci.*, DOI 10.1002/advs.202205485

Ultraelastic and High-Conductivity Multiphase Conductor with Universally Autonomous Self-Healing

Jarkko Tolvanen, Mikko Nelo, Heidi Alasmäki, Tuomo Siponkoski, Piia Mäkelä, Timo Vahera, Jari Hannu, Jari Juuti and Heli Jantunen*

Supporting Information

Ultra-elastic and high-conductivity multiphase conductor with universally autonomous self-healing

Jarkko Tolvanen, Mikko Nelo, Heidi Alasmäki, Tuomo Siponkoski, Piia Mäkelä, Timo Vahera, Jari Hannu, Jari Juuti, Heli Jantunen

This PDF file includes:

Supplementary Text
Figs. S1 to S28
Tables S1 to S4
Captions to Movies S1 to S5

Other Supplementary Materials for this manuscript include the following:
Movies S1 to S5

Captions to the Supplementary Movies:

Movie S1.
Elastic response of the multiphase conductor film during a fast stretch-release process.

Movie S2.
Large-scale self-healing of multi-layered multiphase conductor after bisected from multiple places with a sharp blade.

Movie S3.
Striking of damaged cylinder-shaped electrically insulating self-healing elastomer sample repeatedly with a hammer (1000 frames per second).

Movie S4.
Shooting at the pristine cylinder-shaped Sylgard 184^{TM} PDMS sample with air rifle (1000 frames per second).

Movie S5.
Striking of damaged cylinder-shaped Sylgard 184^{TM} PDMS sample with a hammer (1000 frames per second)

Supplementary Text

Phase separation and morphology

Evolution of morphology and structure formation is related to the thermodynamic instability. The phase separation is governed by the Gibbs free energy of mixing *(ΔG_m)* denoted as*:*

$$\Delta G_m = \Delta H_m - T\Delta S_m \qquad \text{(Eq. S1)},$$

where ΔH_m is an enthalpy of mixing, T is a temperature and ΔS_m is an entropy of mixing. According to the Flory-Huggin's theory, the ΔG_m for a multicomponent material system with three distinct components can be calculated as:

$$\Delta G_m = RT\left(\frac{\phi_1}{N_1 v_1} ln\phi_1 + \frac{\phi_2}{N_2 v_2} ln\phi_2 + \frac{\phi_3}{N_3 v_3} ln\phi_3 + \left(\frac{\phi_1\phi_2}{v_{12}}\chi_{12} + \frac{\phi_1\phi_3}{v_{13}}\chi_{13} + \frac{\phi_2\phi_3}{v_{23}}\chi_{23}\right)\right) \qquad \text{(Eq. S2)},$$

where ϕ_1, ϕ_2, and ϕ_3 are the volume fractions, N_1, N_2, and N_3 are the number of segments per molecule, v_1, v_2, and v_3 are the segmental volumes, v_{12}, v_{13}, and v_{23} are the average segmental volumes, and χ_{12}, χ_{13}, and χ_{23} are the interaction parameters between the components. However, predicting phase separation of the multicomponent system consisting of three distinct phases, polar solvent and amphiphilic surfactant becomes cumbersome through such calculations. This is because the processing conditions must also be considered.

There exists a concentration dependency on the ΔG_m. Hence, the decomposition process would therefore be dependent on the value of ΔG_m. A negative value would be in a favor of spontaneous phase separation in supersaturated solutions. Hence, a more negative ΔG_m would result in a stronger the phase separation. This was supported by the observation that a lower insulating to conducting phase ratio resulted in a better phase separation. By controlling the mixing time of the third solution, the entropy of mixing changes mainly as water evaporates. Hence, longer mixing times resulted in poorer phase separation. The cross-linking temperature should be of consideration as it affects, for example, the evaporation rate of water and polar solvent present in the multicomponent system. Hence, influence the concentration of the solute and phase separation. However, we carried out the polymerization at relative low temperatures to avoid random chain scission and to have a soft phase with well-defined molecular structure.

It is supposed that during solidification, the less soluble compounds (PEDOT-rich nanofibrils and hard phase) are likely becoming more concentrated at the solute-substrate interface with concentration of the solute increasing. The PEDOT:PSS not only has a high surface energy (in relation to the other phases in the multicomponent blend), but more importantly a tendency to crystallize. The solute-solute interactions would then impact the final phase-separation in the conductor. As a result, PEDOT:PSS and hard phase tend to form dual-rich domains near the bottom of the film close to the substrate. Thus, the soft phase occupies a top surface of the film near the air interface. The phase separation was similar in the electrically non-conducting elastomer without PEDOT:PSS. Nearly all compositions were found to show vertically phase-separated morphologies. The high elastic moduli components occupy a larger proportion of the bottom surface of the film. However, we should point out that this would dependent also on the actual composition, processing conditions and thickness of the deposited films.

Equivalent Takayanagi model

Young's moduli of multicomponent systems, such as polymer blends and phase-separated interpenetrated elastomer networks can be approximated with equivalent models, for example,

by using Takayanagi model. Individual phases are typically presented as series and parallel elements presenting individual phases, with different moduli, and volume fractions in the phase separated elastomer (which occupy a different portion of the 3D network).

We adapted the Takayanagi model for the multiphase conductor with an assumption that the hard phase and PEDOT-rich nanofibrils form dual-rich domains at the top surface of the film. In the equivalent Takayanagi model, Young's modulus (E_T) for the multiphase conductor can be defined as $E_T = \lambda[(\varphi/E_1)+\{(1-\varphi)/E_2\}]^{-1}+(1-\lambda)E_3$; where, E_1, E_2, and E_3 are the Young's moduli for structurally stable network (consisting of the hard phase), electrically conducting phase (PEDOT-rich nanofibrils), and dynamic network (consisting of the soft phase), λ and φ are the corresponding volume fraction(s) for the individual phase(s) in the co-continuous 3D network.

To calculate the theoretical Young's modulus, with the equivalent model, we suppose that a volume fraction of the PEDOT-rich nanofibrils can be approximated by assuming that all of the water and most of the secondary dopant are evaporated during solidification. This leads to approximated volume fractions of ≈0.1 - 0.6 vol.% for the PEDOT-rich nanofibrils (with insulating to conducting phase ratios of 10:1 - 2:1). We measured the Young's modulus for the individual phases by then preparing pristine soft phase, hard phase, and PEDOT-rich nanofibril film with Triton™ X-100.

Young's modulus was approximately 1.3, 2.0 and 0.01 MPa for the individual phases. The equivalent Takayanagi model gives theoretical values that vary between 0.195 - 0.201 MPa (depending on the composition). We measured Young's moduli of the multiphase conductors, (with similar composition), to be in the range of 0.136 - 0.160 MPa with extension speed of 10 mm s^{-1} (corresponds to a strain rate of 142.8%s^{-1}).

Preparation of cylinder-shaped samples and conductive coating:
Two distinct types of cylinder-shaped samples were prepared from the developed electrically non-conductive self-healing elastomer and commercial Sylgard 184™ poly(dimethylsiloxane). Multiphase conductor films were prepared by tape-casting the third solution onto a polyethylene terephthalate (PET) film. The conductive film was then transferred from the PET film to a cylinder-shaped sample by rolling the sample over the coated PET film. Due to strong chemical interactions between the conductive coating and the elastomer, the conducting films can be easily transferred to the self-healing elastomer from the PET film. Then, the conductive films would be permanently bonded to the cylinder-shaped elastomer.

Piezoresistive sensing
The water drop height (h) was defined to be approximately 35 mm. The average mass (m) of the water droplet was ≈0.05 grams. The impact force (F) was calculated to be $\approx 9.81\cdot 10^{-4}$ Newtons ($F = mv^2/h$), where v is the velocity in meters per seconds. For a free fall, the v was calculated from the fall time (t) $=\sqrt{(2s/g)}$, where s and g are the fall time and the gravitational acceleration (≈ 9.81 m/s^2), respectively. The contact area of the water droplet was approximated to be $3.41\cdot 10^{-5}$ m^2. This gives a force per area of ≈28.73 Pa for the impact of water droplet. Note that that mass and contact area was overestimated which would mean the corresponding force per area could be considerably lower and the calculated pressure sensitivity better. Thus, the low detection limit for pressure would be lower than 30 Pa without any further compositional modifications.

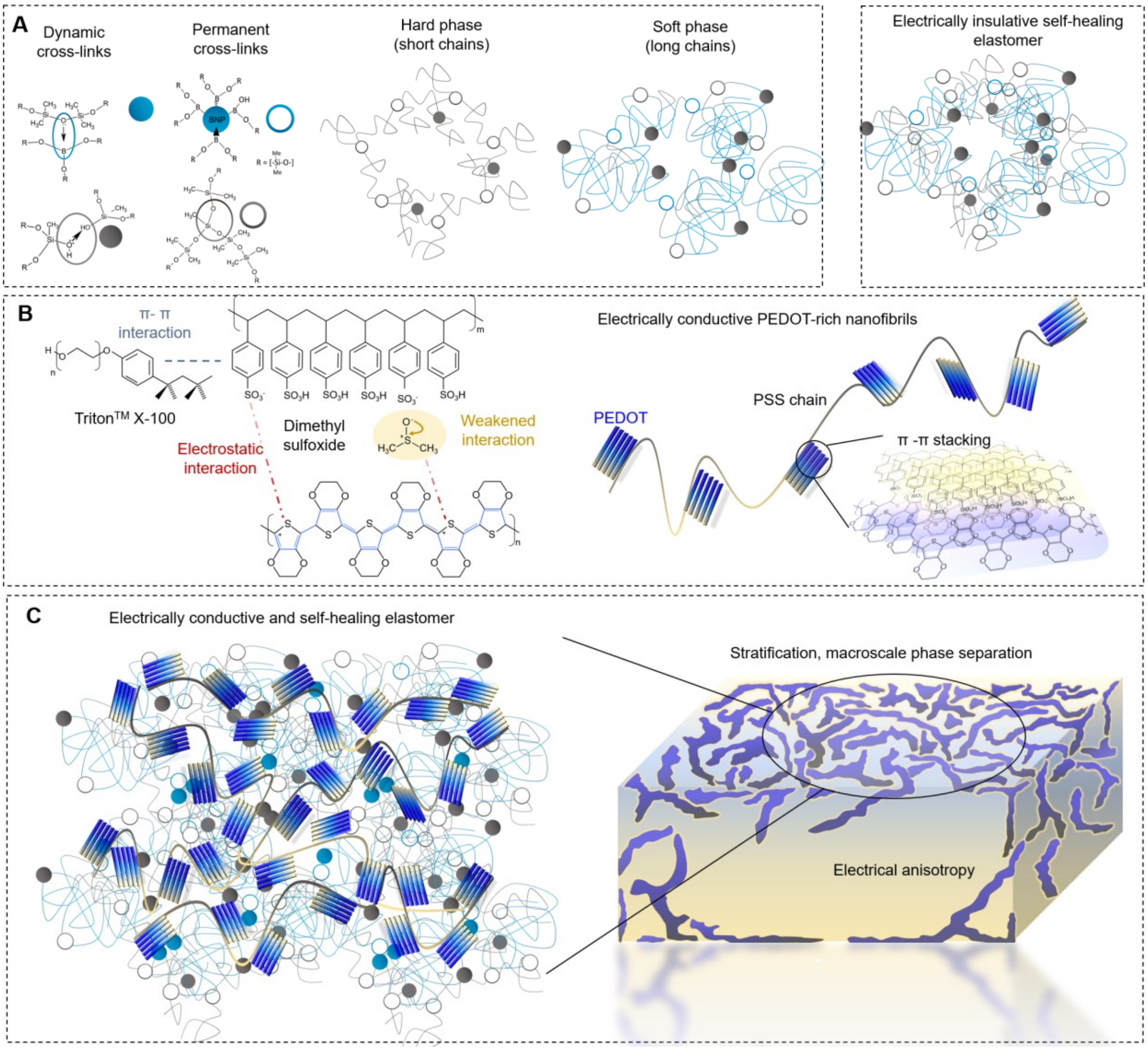

Figure S1. Schematic illustrations of the individual phases and chemical structures.

(**a**) The electrically non-conductive self-healing elastomer consist of soft and hard phases with long and short polymer chains, respectively. As shown , the chemical structures were composed of both covalent bonds and dipole-dipole interactions (reference [13] in the manuscript). (**b**) PEDOT:PSS and conductivity enhancer (dimethyl sulfoxide) form PEDOT-rich nanofibrils. The blending of the components was possible with the use of amphiphilic surfactant, such as Triton™ X-100. (**c**) The electrically conductive self-healing multiphase elastomer was then a combination of the structures shown in the (a) and (b). During solidification, a vertically phase-separated interpenetrated network was formed that results in anisotropic electrical properties. In the 3D schematic, the yellow illustrates the soft phase domains while teal/blueish correspond to the domains with the hard phase/PEDOT-rich nanofibrils.

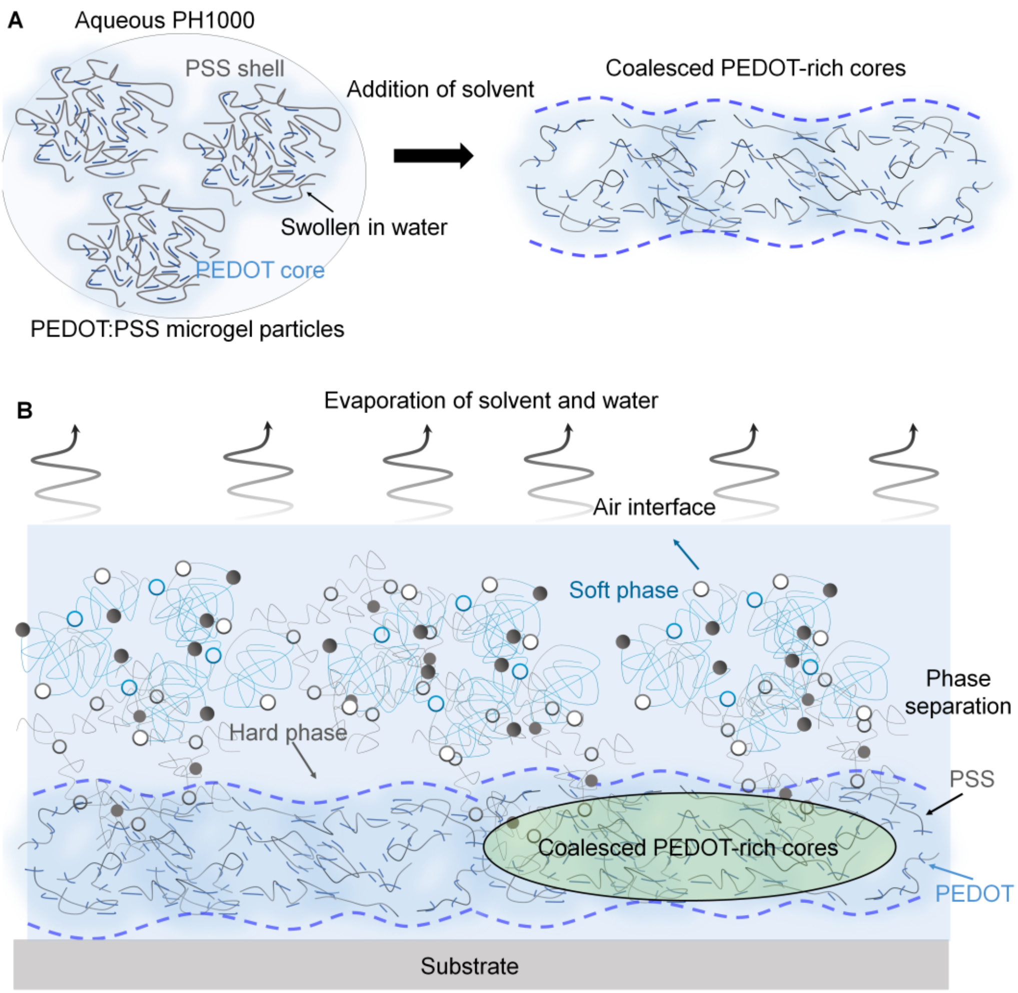

Figure S2. Schematic illustration of the preparation of the multiphase conductor.

(**a**) Aqueous dispersion of PH100 contains PEDOT:PSS microgel particles that are swollen in a water. These microgel particles consist of insulative PSS-shell and conductive PEDOT-core. Addition of polar solvent (such as dimethyl sulfoxide; DMSO) results in coalescing the PEDOT-rich cores. Thus, resulting in a formation of PEDOT-rich nanofibrils at the critical volume threshold of the DMSO (≈16 vol.%). (**b**) During solidification, the less soluble components are becoming more concentrated at the solute-substrate interface with concentration of the solute increasing. Hence, the PEDOT-rich nanofibrils and the hard phase tend to form dual-rich regions near the bottom of the film close to substrate. The soft phase occupies the space close to the air interface. With significantly thinner films, the phase separation was inverse to that described here.

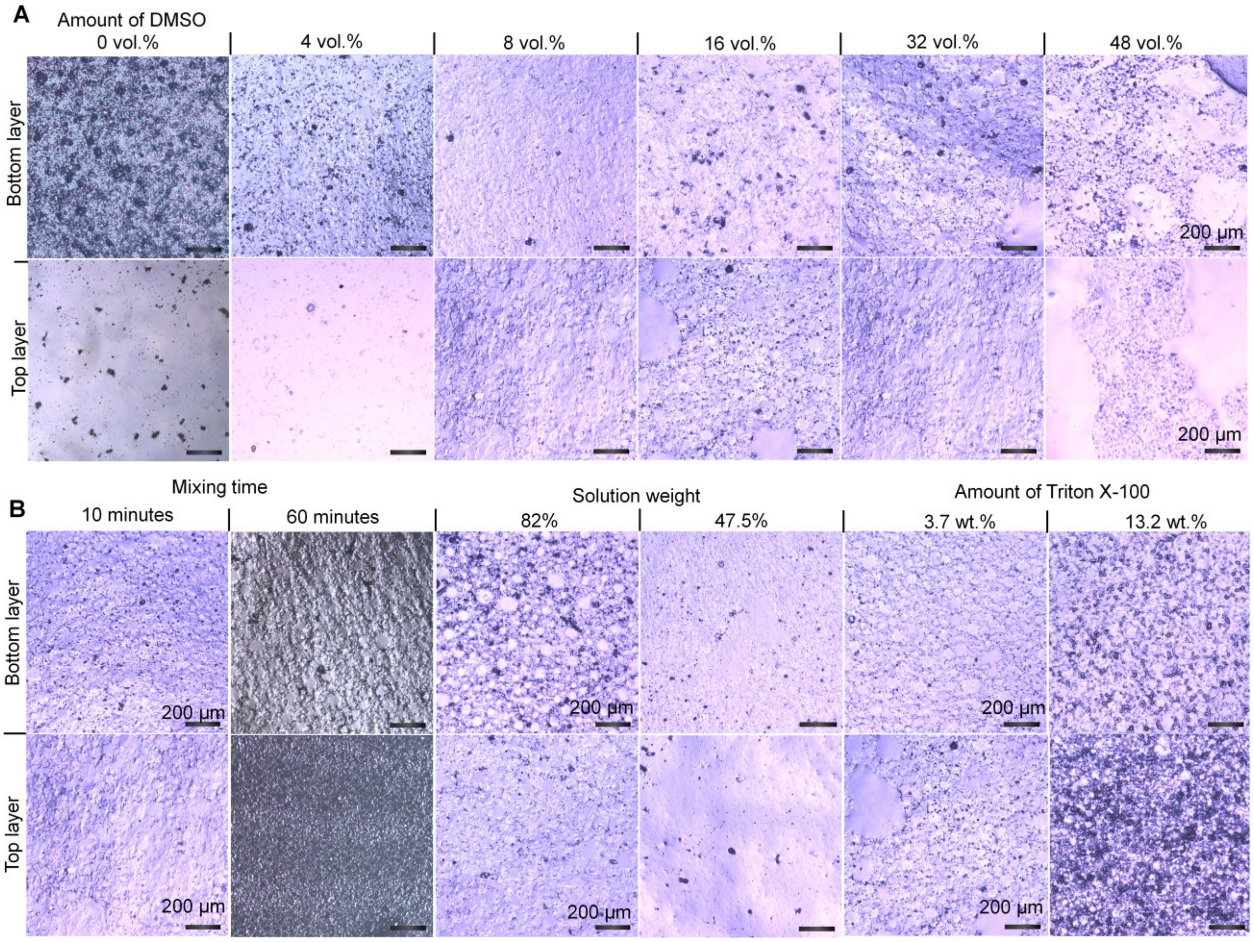

Figure S3. Optical micrographs of multiphase conductors.

(**a**) DMSO content was varied from 0 to 48 vol.%. Other compositional parameters were kept constant: ratio 2:1, X-100 content 1.3 wt.%, the weight of the first solution 59 wt.% and mixing time of the third solution 15 minutes (top row). (**b**) The mixing time of the third solution was 10 minutes and 60 minutes (the bottom row on the left). Other compositional parameters were kept constant: ratio 2:1, DMSO content 16 vol.%, X-100 content 1.3 wt.%, and weight of the first solution 59 wt.%. The weight of the first solution was 82 wt.% and 47.5 wt.% while other parameters kept similar (the bottom row in the middle). Mixing time of the third solution was 15 minutes. X-100 content was 3.7 wt.% and 13.2 wt.%. Other parameters were kept similar (the bottom row on the right).

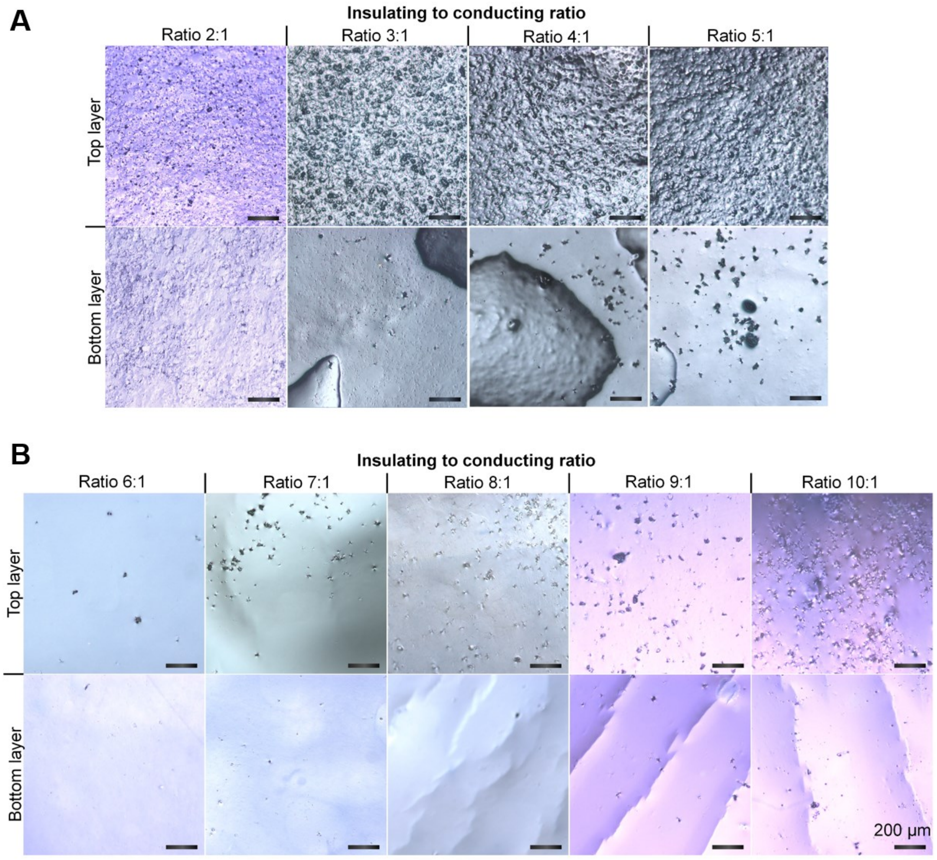

Figure S4. Optical micrographs of multiphase conductors.

Multiphase conductors with varied insulating to conducting phase ratios (from 2:1 to 10:1). Other compositional parameters were kept constant: DMSO content 16 vol.%, X-100 content 1.3 wt.%, weight of the first solution 59 wt.%, and mixing time of the third solution 15 minutes. The adhesion to substrate increases with the ratio increasing. This was visible as lines in the optical micrographs which indicate transverse compression when the films are peeled off from the substrate for optical imaging.

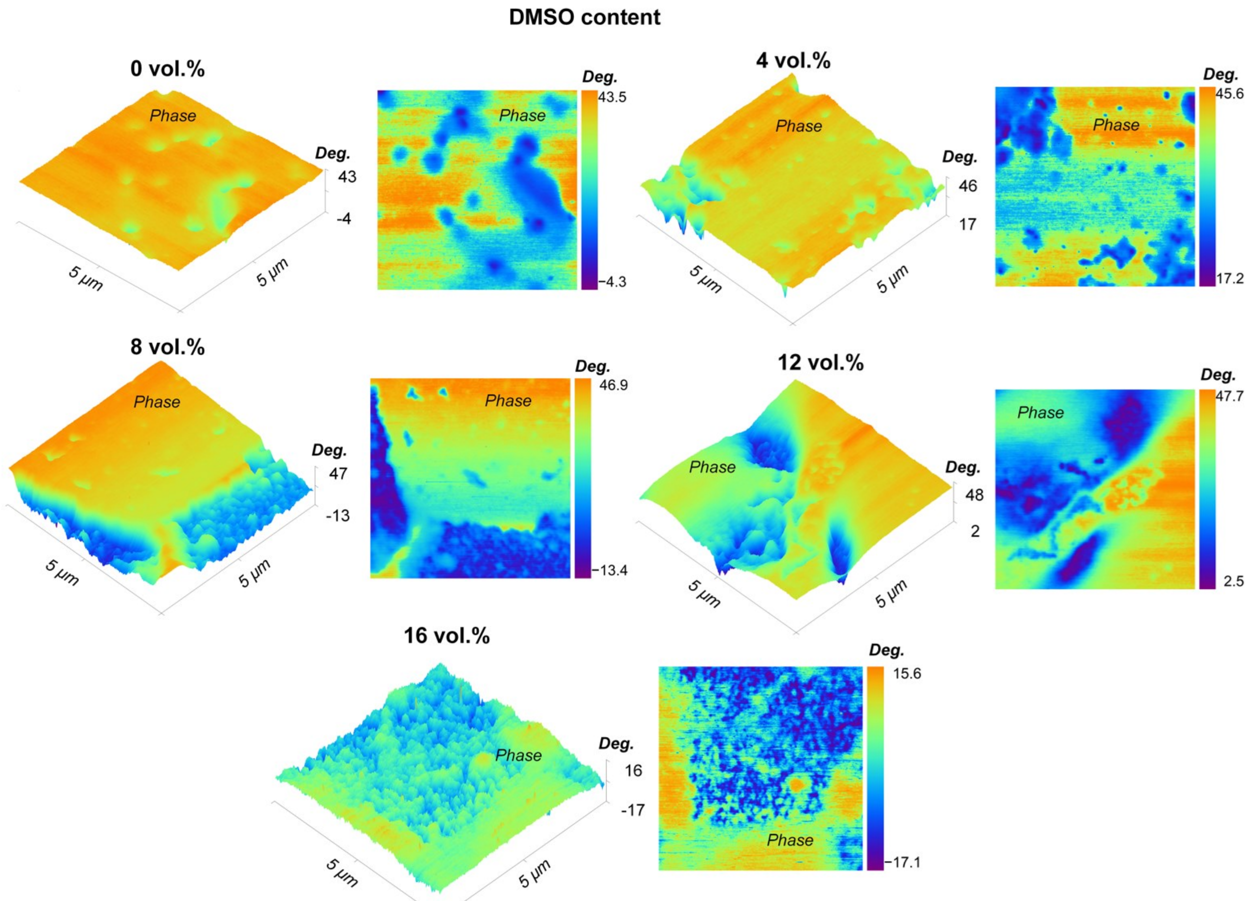

Figure S5. 2D and 3D phase AFM images with varied DMSO content.

2D and 3D phase AFM images measured in tapping mode from the multiphase conductors with varied DMSO content (from 0 vol.% to 16 vol.%). Other compositional parameters were kept constant: ratio 2:1, X-100 content 3.7 wt.%, weight of the first solution 59 wt.%, and mixing time of the third solution 15 minutes.

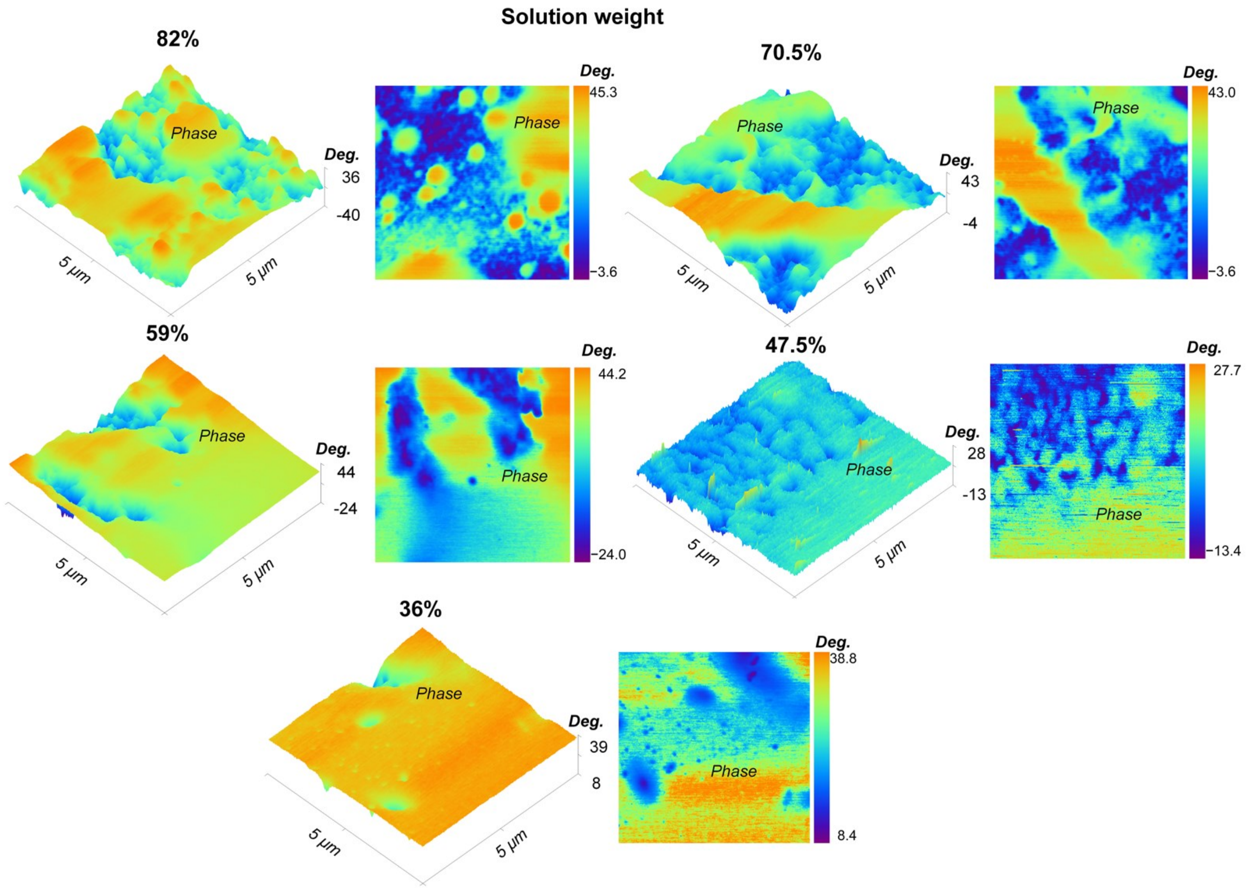

Figure S6. 2D and 3D phase AFM images with varied weight of the first solution.
2D and 3D phase AFM images measured in tapping mode from the multiphase conductors with varied weight of the first solution (from 82 wt.% to 36 wt.%). Other compositional parameters were kept similar than in Figure S6.

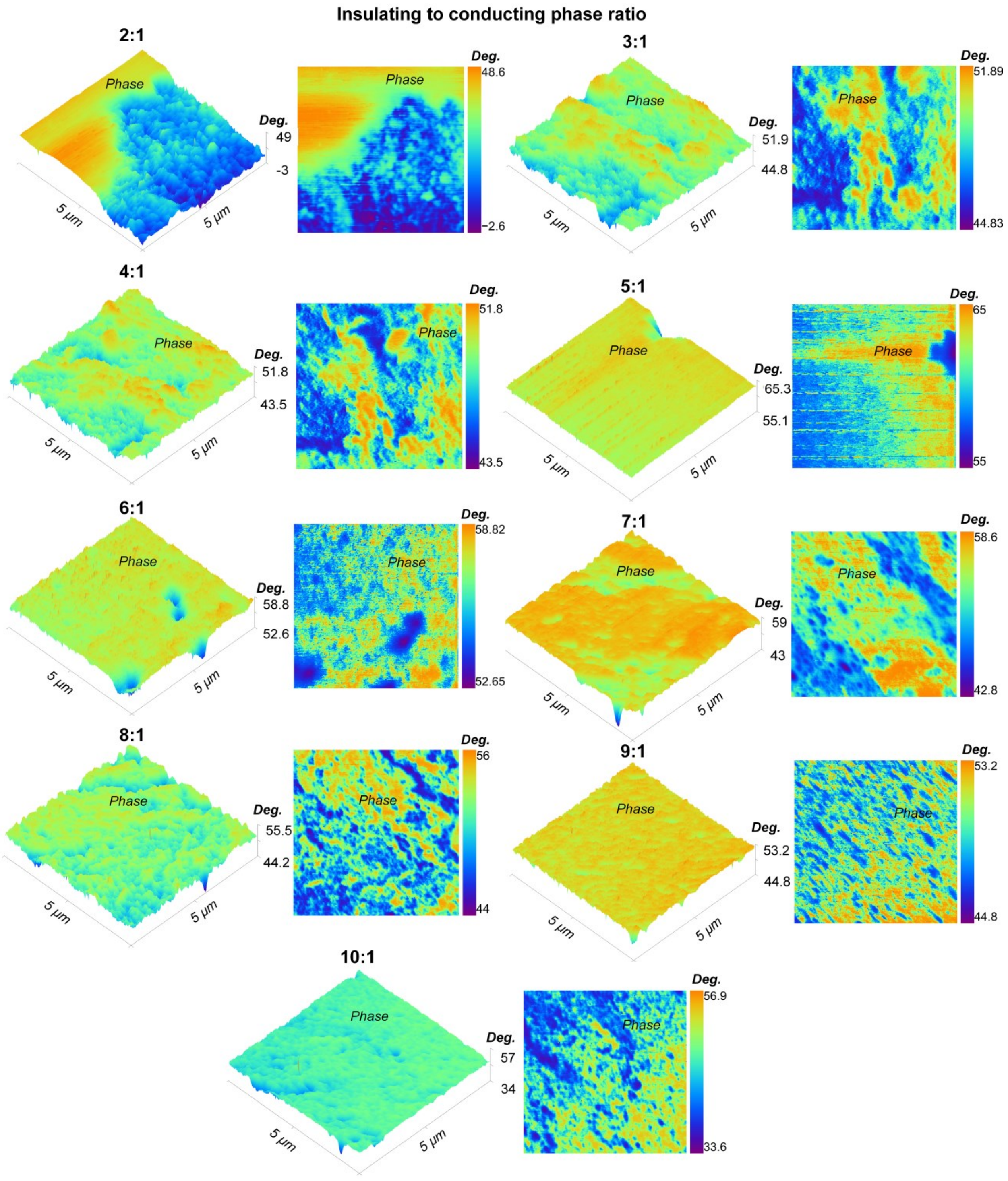

Figure S7. 2D and 3D phase AFM images with varied insulating to conducting phase ratios.

2D and 3D phase AFM images measured in tapping mode from the multiphase conductors with varied insulating to conducting phase ratios (from 2:1 to 10:1). Other compositional parameters were kept similar: DMSO content 16 vol.%, X-100 content 3.7 wt.%, weight of the first solution 59 wt.%, and mixing time of the third solution 15 minutes.

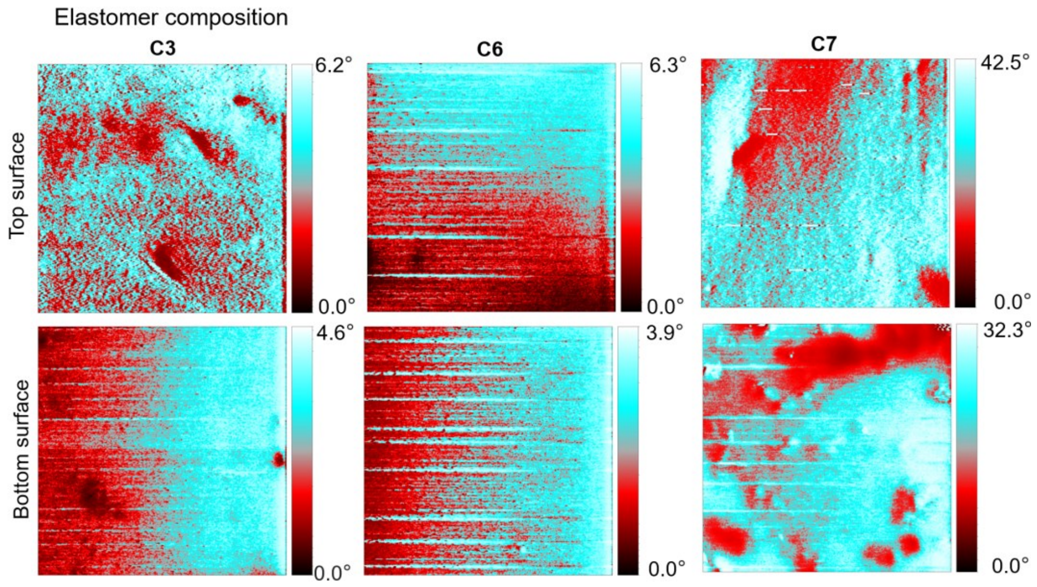

Figure S8. 2D phase AFM images of electrically insulating elastomers.

Non-conductive self-healing elastomer compositions denoted as C3, C6, and C7 (see reference [13] in the manuscript). The difference between C3 and C6 elastomers was the cross-linking temperature (120 °C vs. 70 °C). The ratio of polymer base to cross-linking component was 10:1. The difference between C6 and C7 elastomers was the ratio (10:1 vs 5:1). The heterogeneity and phase separation changes with the composition which improves the tensile properties.

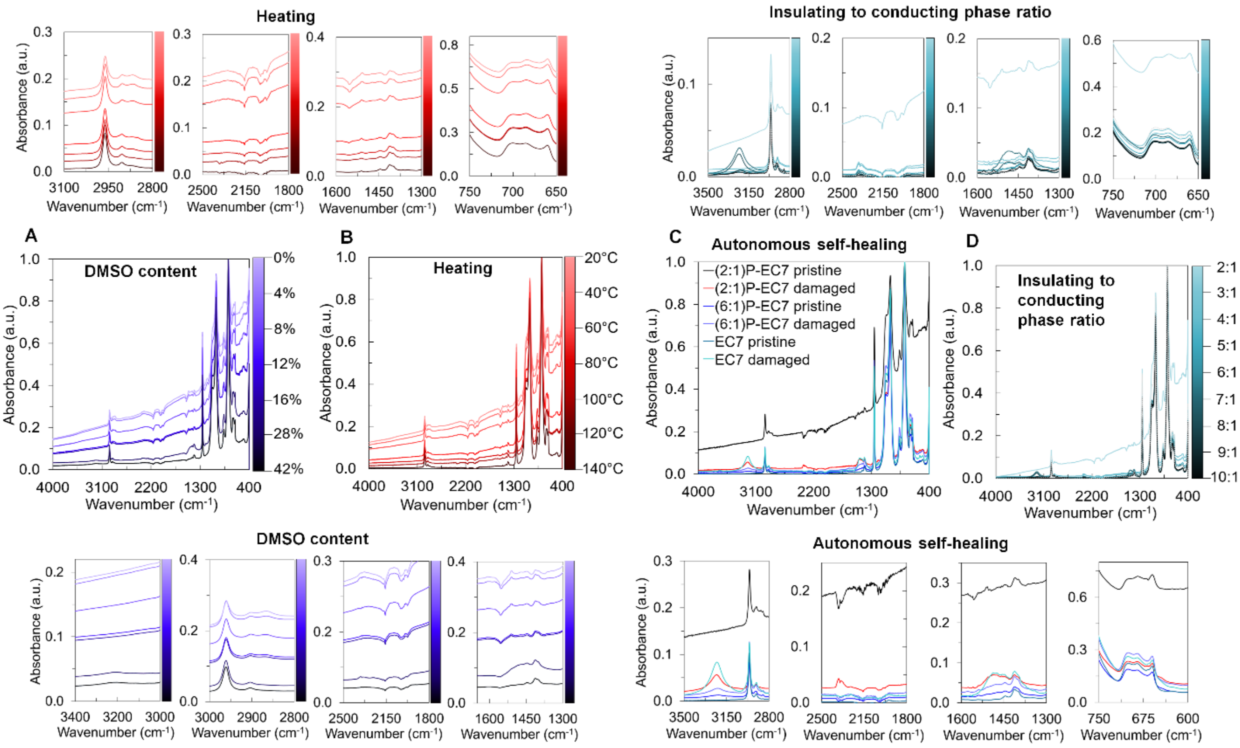

Figure S9. FTIR spectras for multiphase conductors and elastomers.

(**a**) Pristine multiphase conductors with varied DMSO content. (**b**) Temperature-dependent FTIR spectras upon heating multiphase conductor from 20 °C to 140 °C. (**c**) FTIR spectras in static state (pristine sample) and dynamic states (after mechanically damaged) for multiphase conductors (denoted as P-EC7) and electrically non-conductive self-healing elastomer (denoted as EC7). The EC7 refers to C7 composition in our previous work. (**d**) Multiphase conductors with varied insulating to conducting phase ratios (2:1 to 10:1).

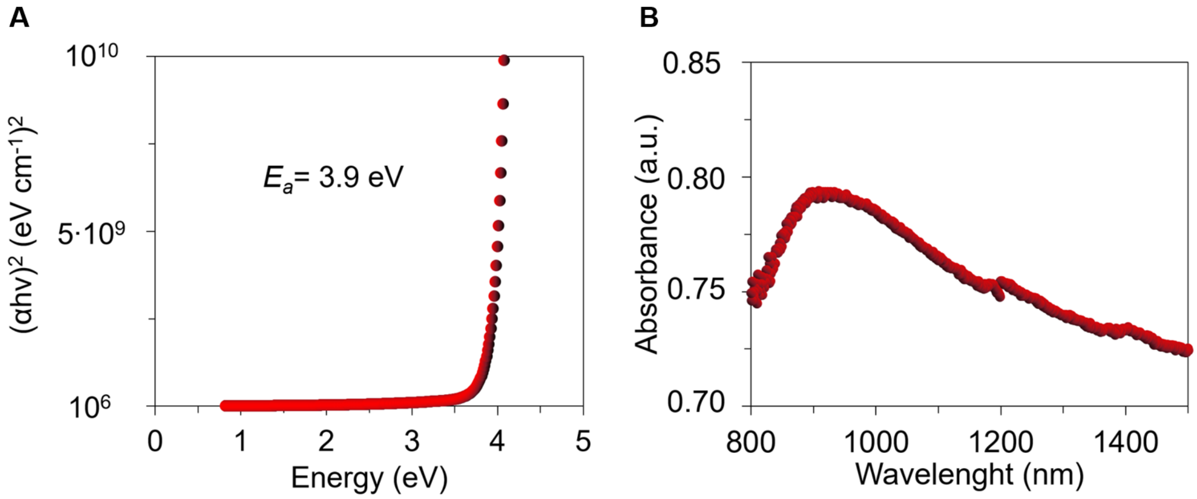

Figure S10. (a) Bandgap energy plot and (b) absorbance as a function of wavelength.

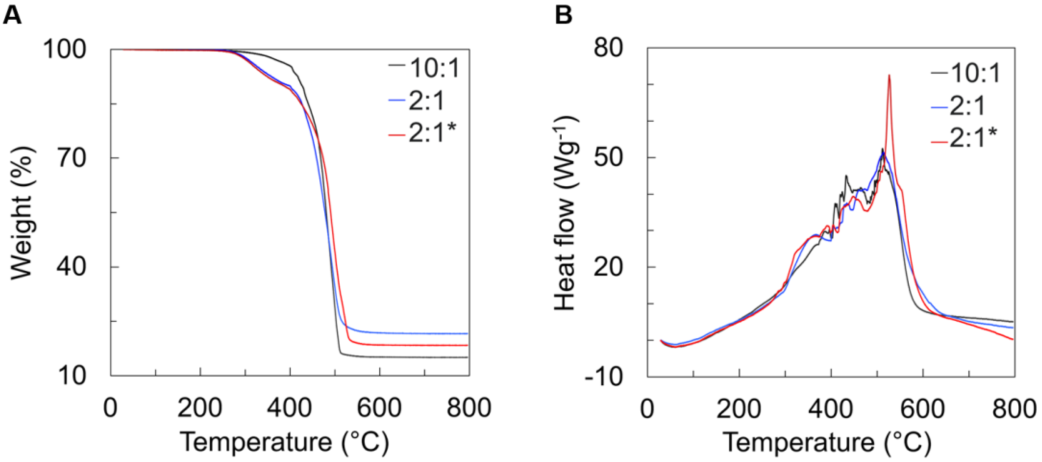

Figure S11. (a) Thermogravimetric analysis and (b) differential scanning calorimetry. Multiphase conductors with insulating to conducting phase ratios of 10:1 and 2:1, where * denotes an elastomer without DMSO. Data was collected during the second heating process after eliminating the thermal history. Heating rate was 10 °C min^{-1}. The conductor with 10:1 ratio was kept in ambient conditions until interdiffusion of phases occurred. This improves the temperature stability as the vertical phase separation was suppressed.

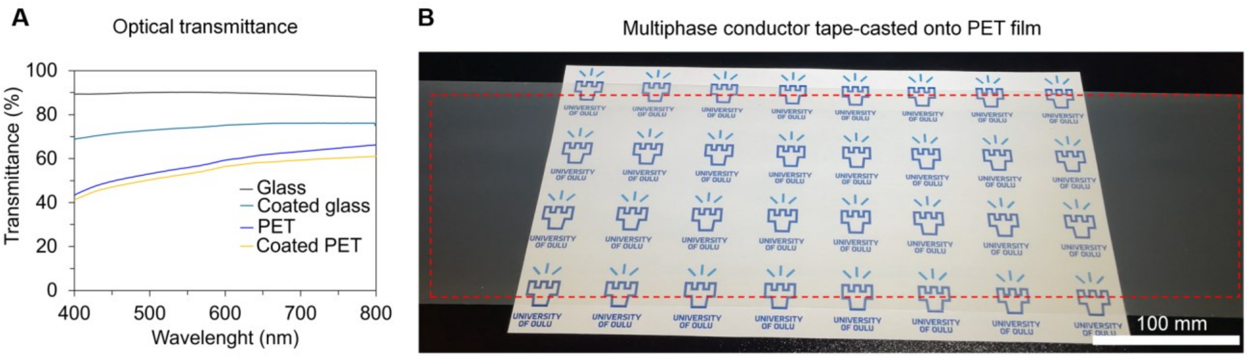

Figure S12. (a) Optical transmittance spectras and (b) photograph of tape-casted film. Optical transmittance spectras for multiphase conductors with insulating to conducting phase ratio of 2:1. Thicknesses of the films were ≈25 µm. Photograph of the multiphase conductor film tape-casted onto PET film. Thickness of the film was ≈25 µm.

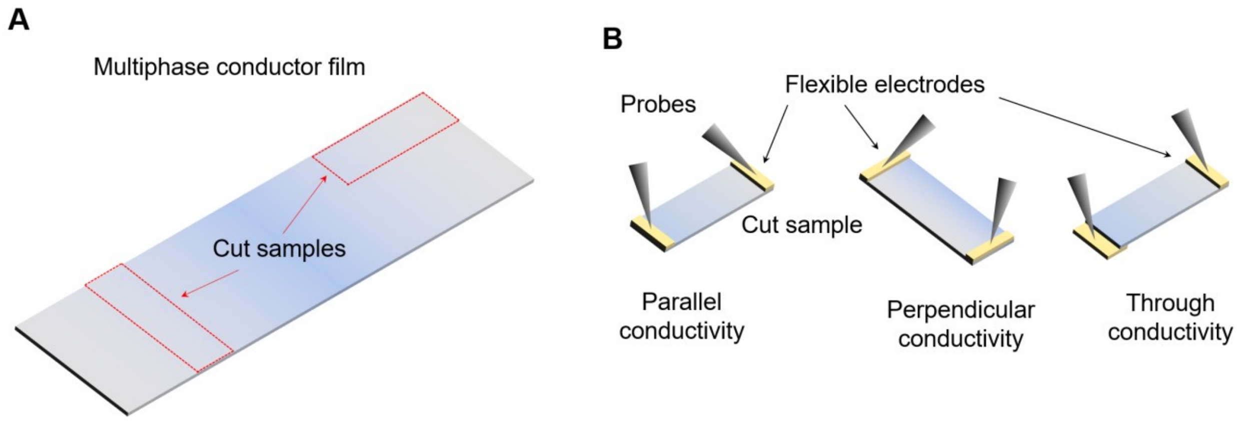

Figure. S13. Schematic illustrations (a) how samples were cut and (b) how the measurement probes were connected to the cut samples.

The conductivity from the top surface of the film was measured in similar manner than from the bottom side of the film (parallel conductivity). It should be noted that the conductivity was less than 10^{-5} S cm^{-1} for the side of the film that faced the air interface (please see Figure S2). The surface of a film that faced against the substrate had significantly better electrical conductivity. Hence, the films were inverted for the measurements before peeling off the substrate and attaching the flexible electrodes. The conductivity in the poorly conducting side of the film was lower than the through conductivity for compositions that showed $\sigma_{z\text{-}axis}$ values over 10^{-5} S cm^{-1} (please see Figures S15 and S16).

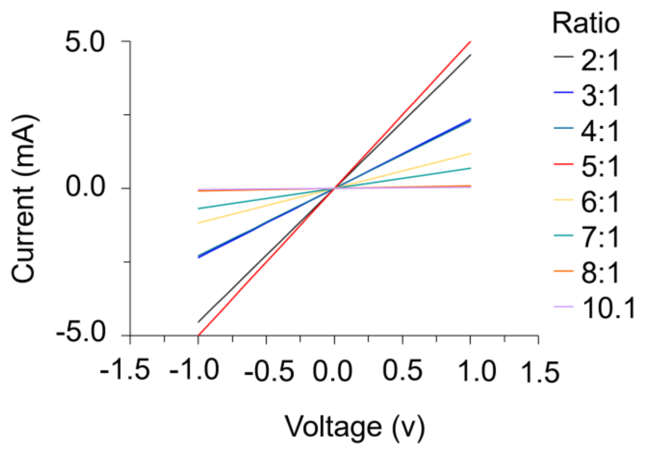

Figure S14. I-V characteristics of multiphase conductors.

I-V characteristics when sweeping from -1.0 V to 1.0 V. The insulating to conducting phase ratios were varied from 2:1 to 10:1. Other compositional parameters were kept constant: DMSO content 16 vol.%, X-100 content 1.3 wt.%, weight of the first solution 59 wt.%, and mixing time 30 minutes.

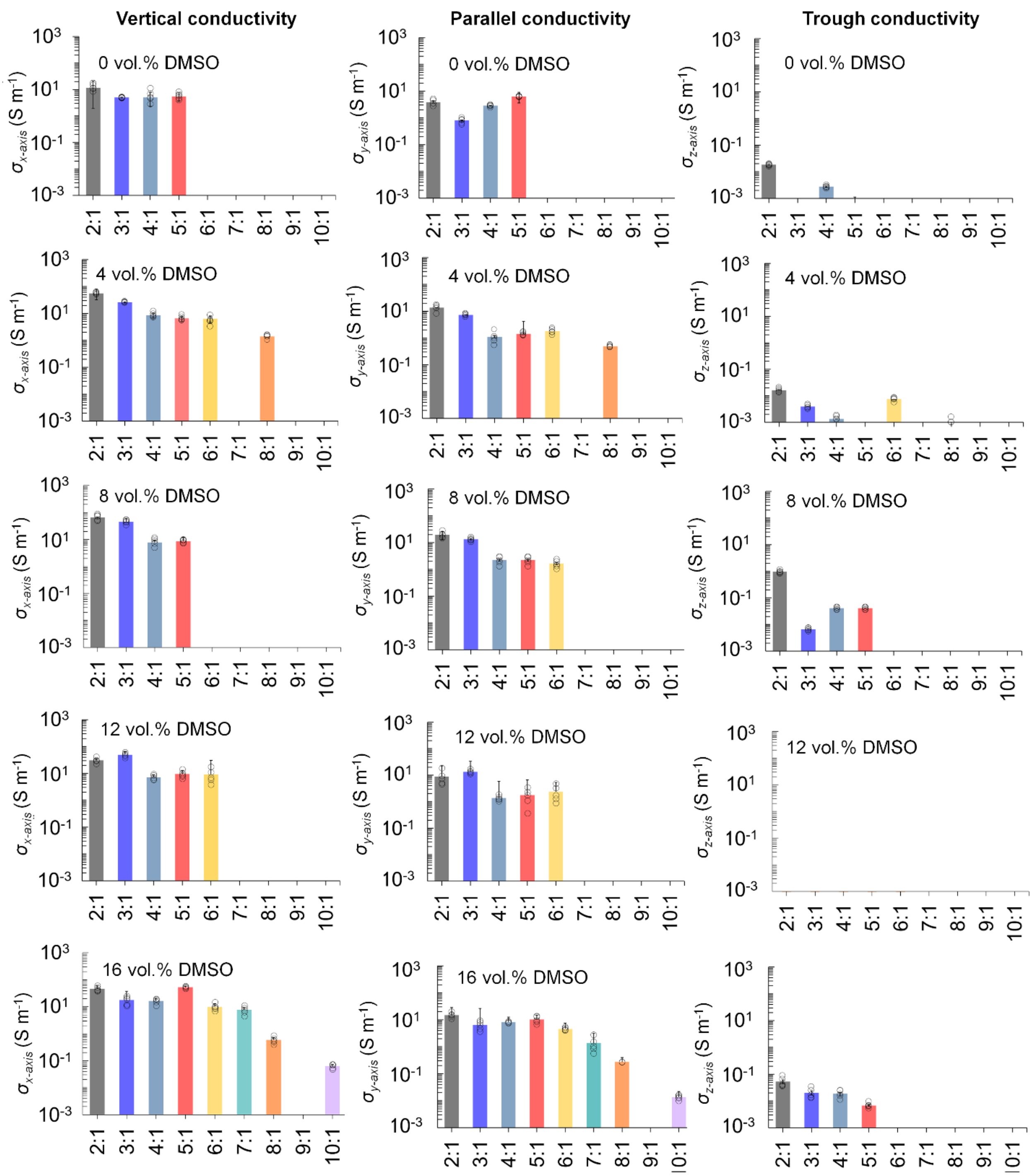

Figure S15. Direct current electrical conductivities.

Conductivities as a function insulating to conducting phase ratios and DMSO content. Other compositional parameters were kept constant: X-100 content 1.3 wt.%, weight of the first solution 59 wt.%, and mixing time of the third solution 30 minutes. The measurements were conducted immediately after the cross-linking was complete. Data expressed as a mean ± SD (n = 5). Individual measurement points shown as non-filled dots. Note that the mean values were used for the contour plots shown in the manuscript. For other compositions, the conductivity was less than 10^{-5} S cm^{-1}.

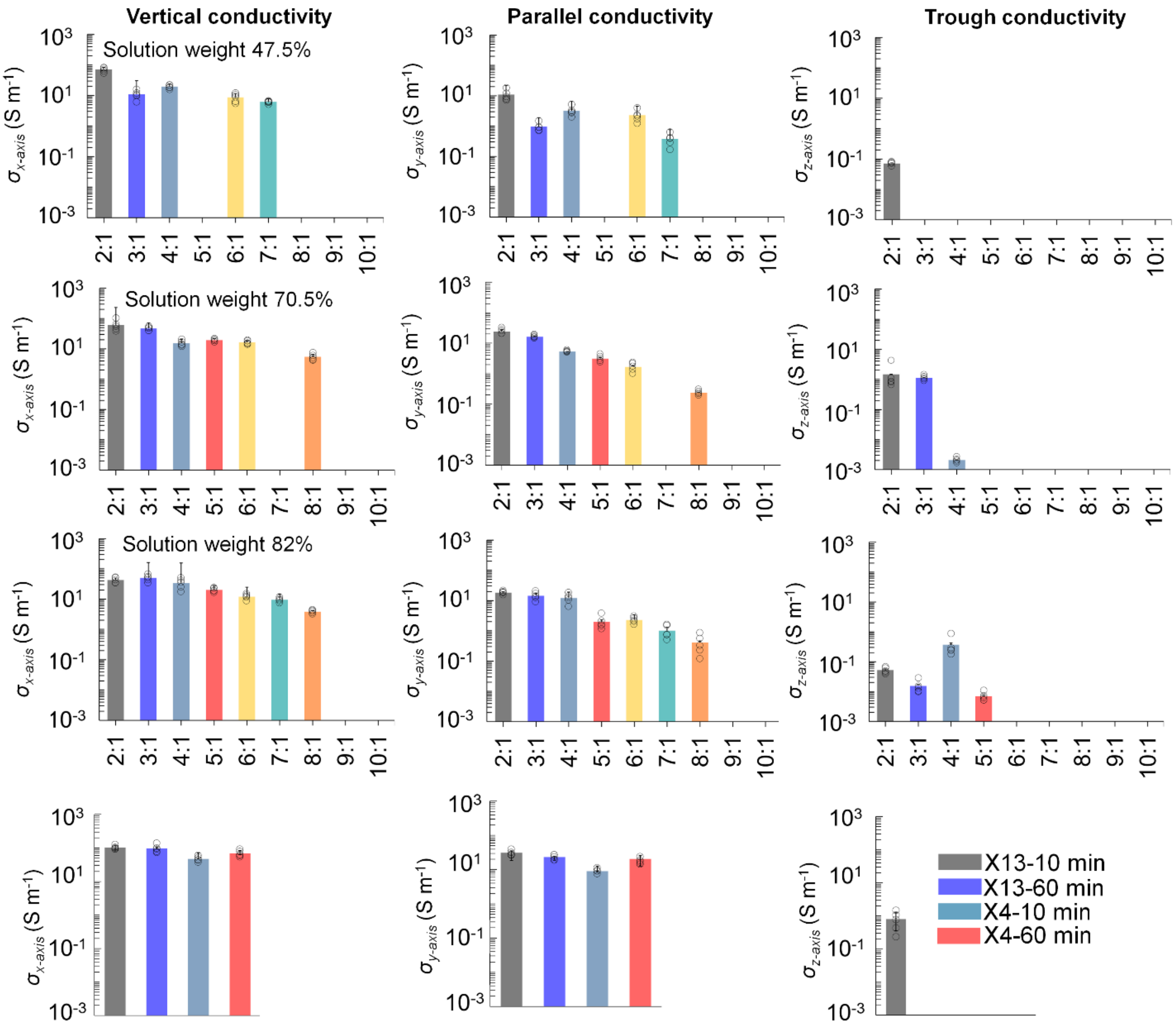

Figure S16. Direct current electrical conductivities.

Conductivities as a function of insulating to conducting phase ratios and weight of the first solution. Other compositional parameters were kept constant: DMSO content 16 vol.%, X-100 content 1.3 wt.%, and mixing time of third solution 30 minutes. The measurements were conducted immediately after the cross-linking was complete. Data expressed as a mean ± SD (n = 5) Individual data points shown as non-filled dots. Note that the mean values used for the contour plots shown in the manuscript. For other compositions, the conductivity was less than 10^{-5} S cm^{-1}.

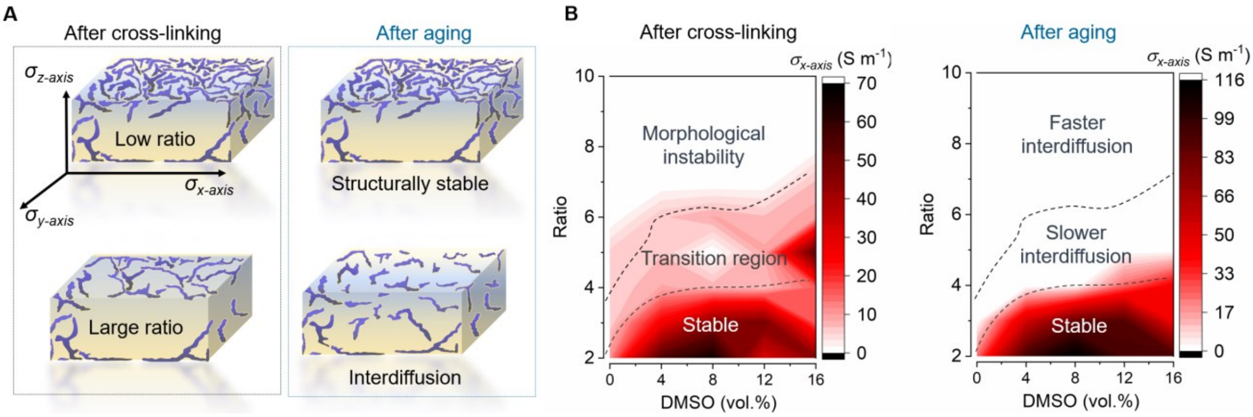

Figure S17. (a) Schematic illustration of morphological stability of the conductors and (b) parallel conductivity after cross-linking and aging.

(**a**) Schematic illustration of morphologically stable (top row) and instable (bottom row) multiphase conductors. Interdiffusion of phases occurs during the aging especially with large insulating to conducting phase ratios. This results in loss of electrical conductivity and anisotropy. (**b**) Parallel conductivity (expressed as $\sigma_{x\text{-}axis}$) plotted after cross-linking and aging for approximately 20 days in ambient conditions. The interdiffusion of phases was slower in the transition region than in the region of morphological instability. The conductivity increases over time during the aging for stable compositions after the cross-linking.

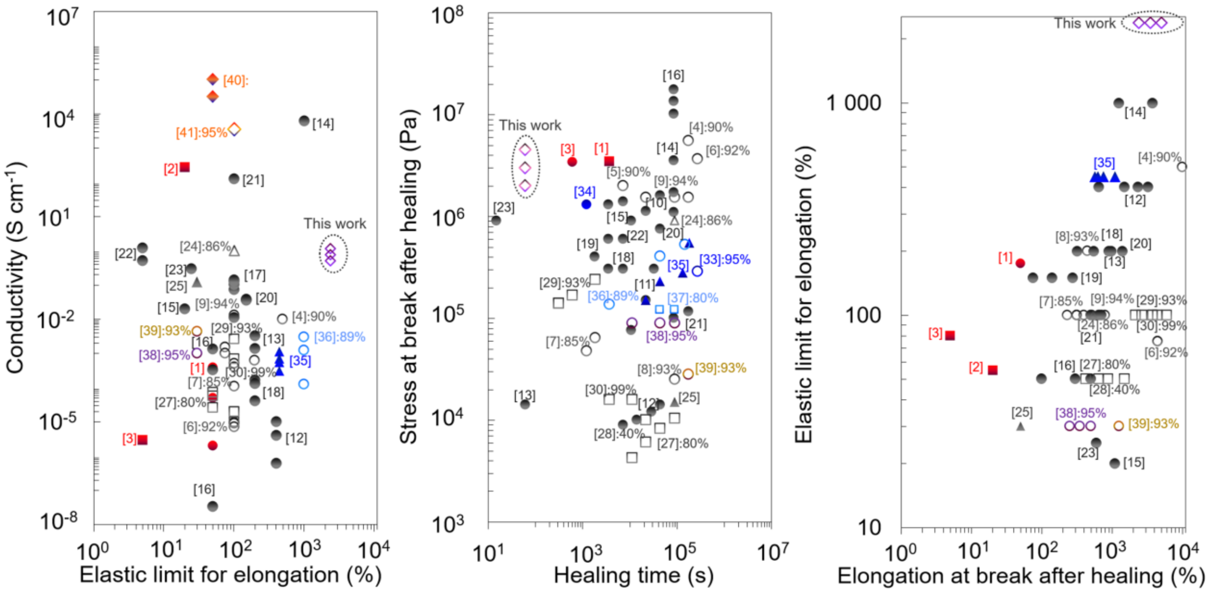

Figure S18. Material property comparisons.

Material properties were compared to the state-of-the-art conductors capable of autonomously self-healing or upon external intervention. Transparent and non-transparent conductors are denoted with non-filled and filled data points, respectively. Optical transmittance was given at 550 nm wavelength as a single value. Hydrogen bonds, metal-ligand interactions and other (such as ionic and combined effects) are denoted by circles, triangles, and squares, respectively. Color coding for self-healing materials are as follows: external energy input trigger required (red), autonomous self-healing in room temperature (less than 40 °C) (grey), cold conditions (below 0 °C) (dark blue), underwater (light blue), various pH values (brown), and universal conditions (purple). Materials classified as universally autonomous self-healing materials were defined as those capable of self-healing in at least five different dry and wet conditions. Elastic limit for elongation was given as maximum uniaxial elongation from which the materials could either fully recover, or to a point where small residual strain existed. Electrical conductivities (S m^{-1}) are taken from pristine undamaged conductors or calculated based the provided information (if not directly given in the references). Note that references [40, 41] are non-healable conductors. List of references for the figures are given in Table S1.

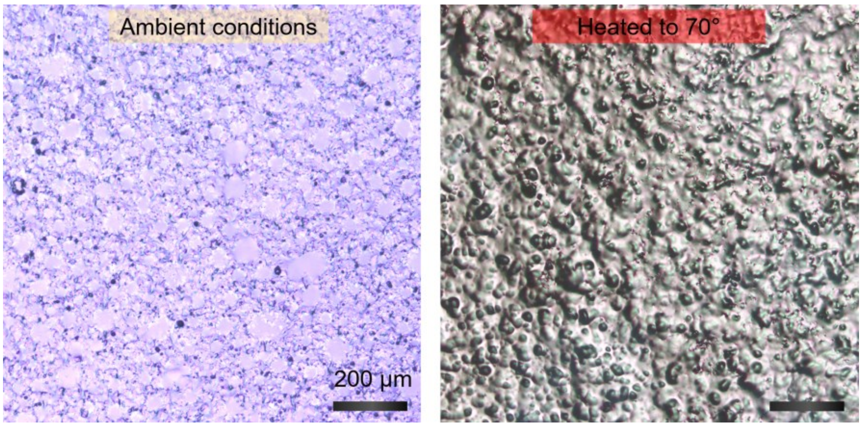

Figure S19. Optical micrographs.

Optical micrographs of multiphase conductor when kept in ambient conditions and upon heating from 20 °C to 70 °C. The heating process was reversible and can be repeated multiple times. The smooth areas in the optical micrograph (on the left) correspond to the soft phase.

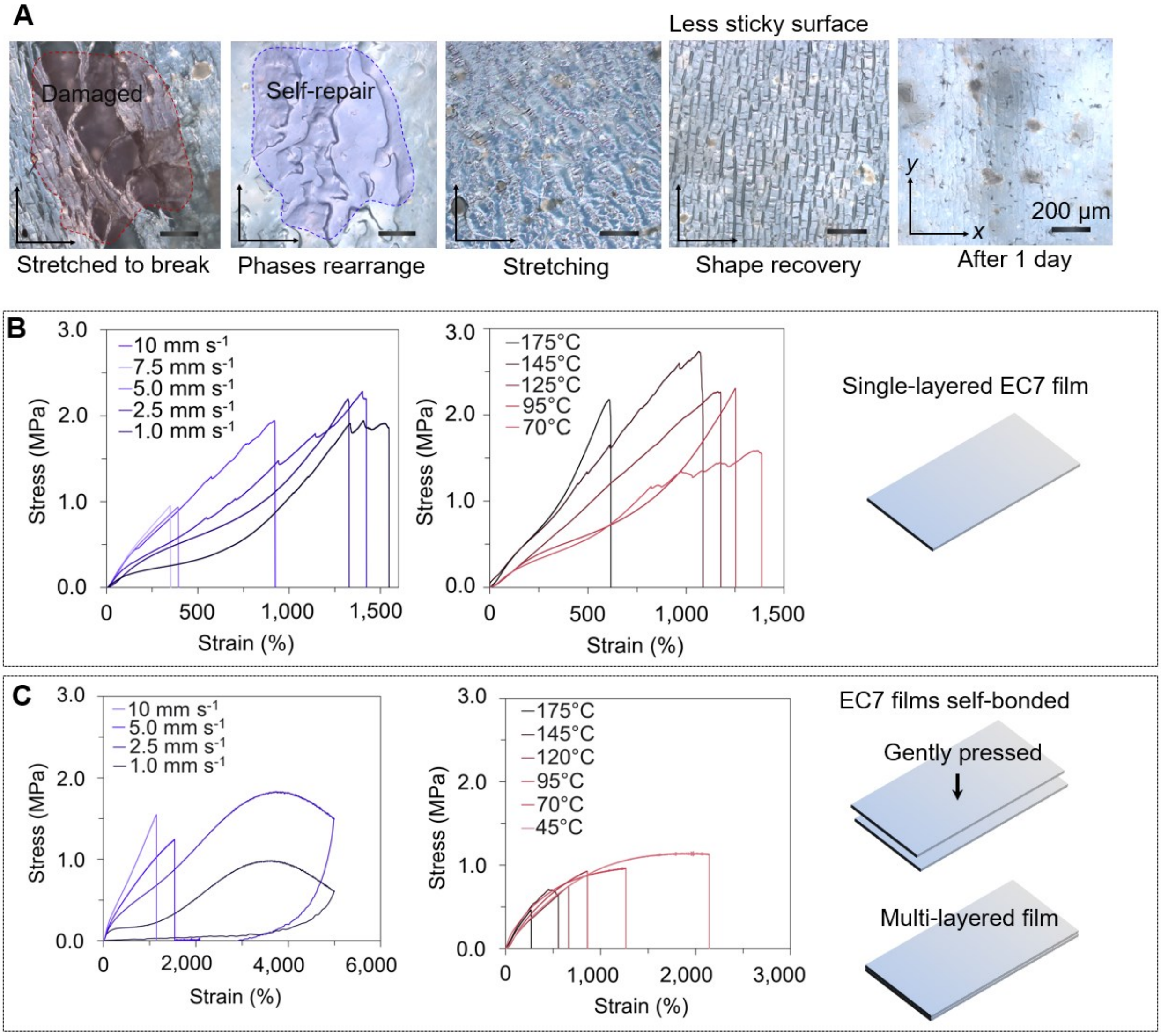

Figure S20. (a) Optical micrographs and (b-c) stress-strain curves for electrically insulating self-healing elastomer.

(**a**) Pristine, damaged, and stretched electrically non-conductive self-healing elastomer. The fractures present in the elastomer eventually dissappear over-time as the phases rearrange themselves. The symmetrical out-of-plane surface structures only appear to film surface that was against the substrate during cross-linking. The surface was also found to be less adhesive. The total spacings between the surface structures corresponded to the existing residual strain in the film during the shape recovery. The surface structures fully dissappear after 1 day in ambient conditions. Stress-strain curves for (**b**) single-layered and (**c**) multi-layered electrically insulating elastomer films as a function of extension speed (left) and upon heating (right). The extension speeds correspond to strain rates of 14.28 $\%s^{-1}$- 142.8 $\%s^{-1}$. The multi-layered films were prepared by gently pressing two single-layered films together. The films permanently bond together and cannot be separated anymore.

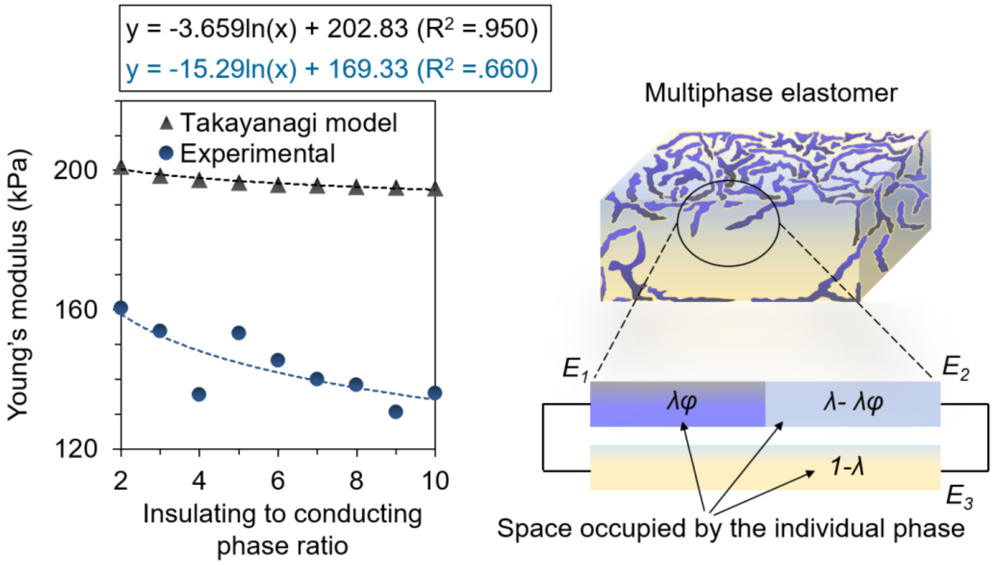

Figure S21. (a) Theoretical and experimentally defined Young's modulus and (b) schematic illustration of space occupied by the individual phases.

The volume fraction occupied by the individual phases are given as a function λ and φ by assuming that the phases may be continuous or discontinuous within the 3D elastomeric network. The multiphase conductor was a single-layered and self-stratified. The hard phase and PEDOT:PSS mostly occupy the top layer of the film (i.e., series elements). The soft phase has been either distributed or interdiffused to the other side of the film (i.e., parallel element). Thus, the volume fractions for individual phases are now given as $\lambda\varphi$, λ-$\lambda\varphi$, and 1-λ, respectively.

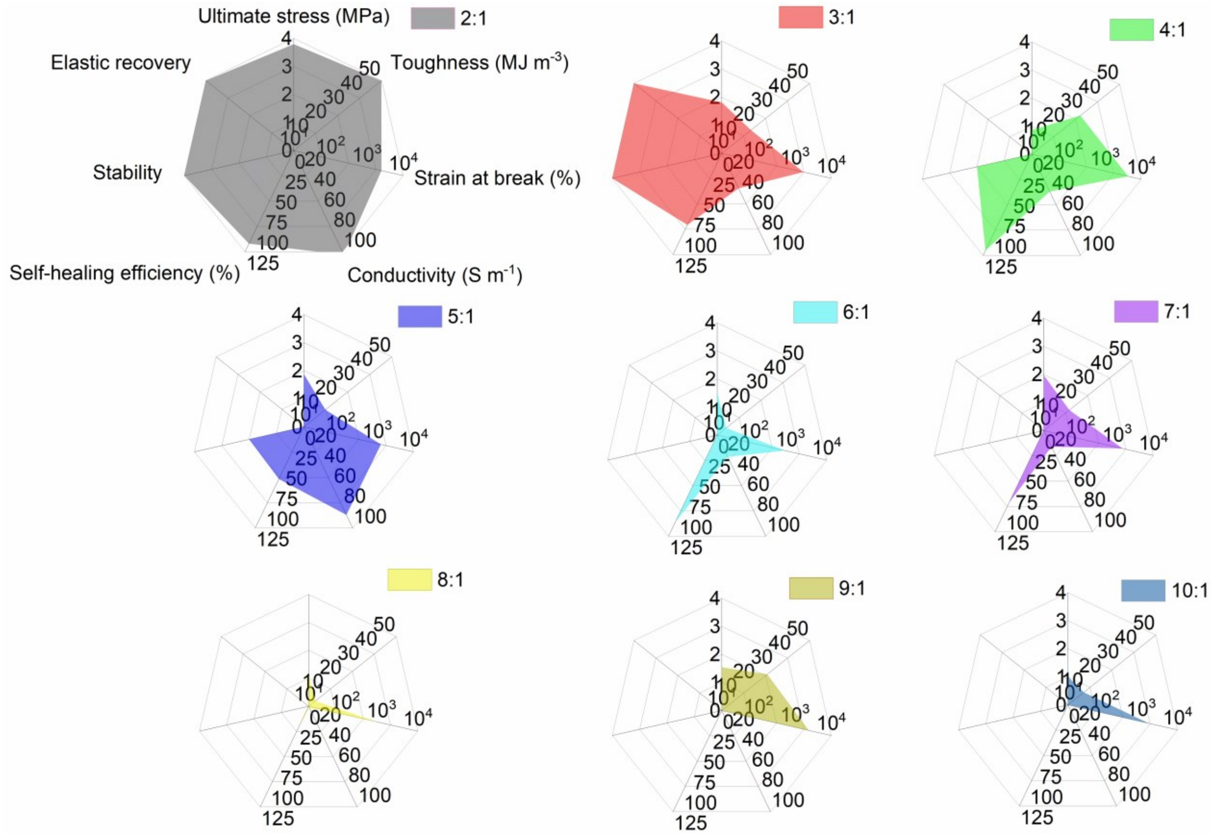

Figure S22. Radar plot for comparison of multiphase conductors.

Multiphase conductors with varied insulating to conducting phase ratios (2:1 to 10:1). Ultimate stress (MPa), toughness (MJm^{-3}), strain at break (%), electrical conductivity (Sm^{-1}), self-healing efficiency (%), morphological stability, and elastic recovery from large deformation were compared. Note that tensile properties were plotted with strain rate of 285% s^{-1}. Other compositional parameters and processing conditions were kept constant: DMSO content 16 vol.%, X-100 content 1.3 wt.%, weight of the first solution 59 wt.% and mixing time of the third solution 15 minutes. Data expressed as mean values (n ≥ 3) and the combined figure was shown in Figure 3d (in the manuscript).

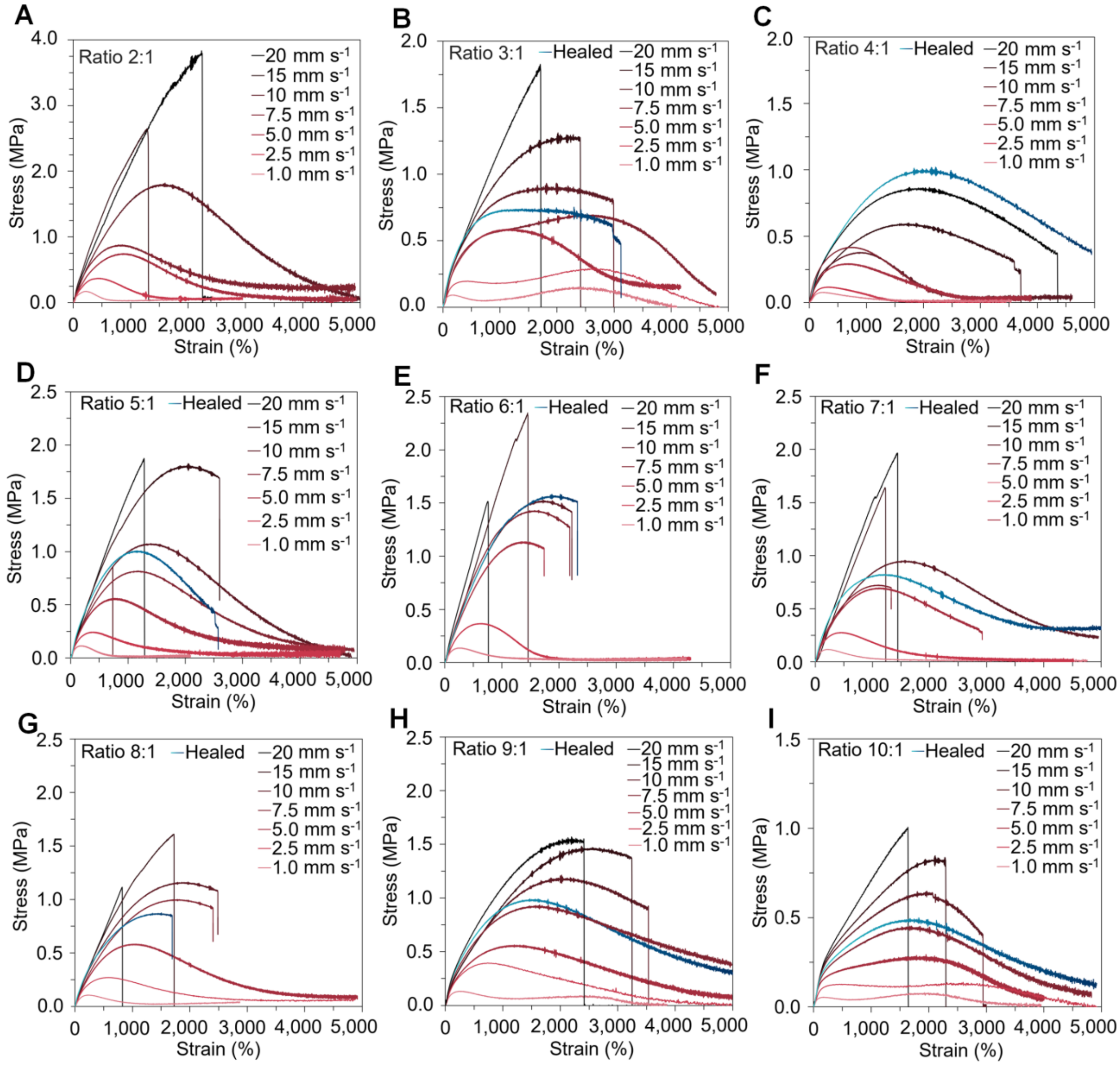

Figure S23. Stress-strain curves for elastomers.

(**a-i**) Tensile properties as a function of extension speed. Insulating to conducting phase ratios were varied from 2:1 to 10:1. The extension speeds correspond to strain rates of 14.28%s^{-1} to 285.7%s^{-1}. The self-healing time was ≈60 seconds after the cut-surface alignment. Other compositional parameters were kept constant: DMSO content 16 vol.%, X-100 content 1.3 wt.%, weight of the first solution 59 wt.%, and mixing time of the third solution 15 minutes.

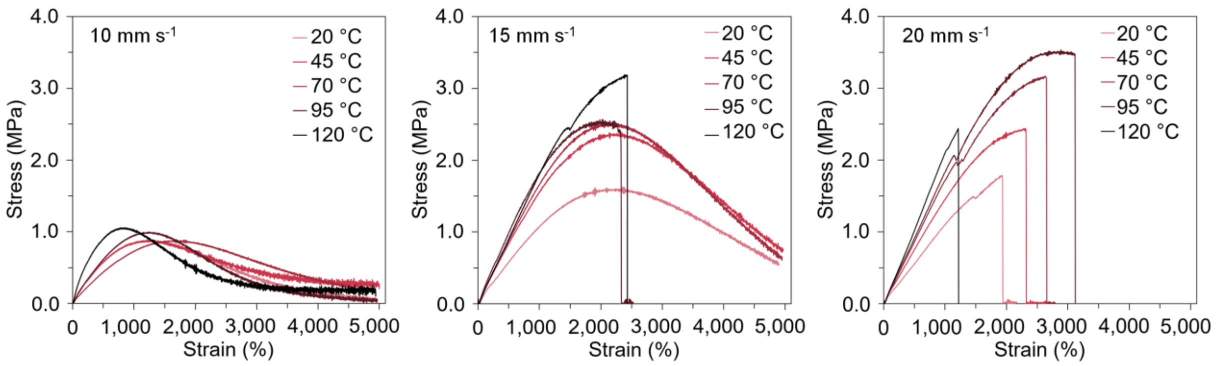

Figure S24. Stress-strain curves for elastomers.

Tensile properties as a function of extension rate and temperature with multiphase conductors when the insulating to conducting phase ratio was 2:1. The extension speeds correspond to strain rates of 14.28%s^{-1} to 285.7%s^{-1} Other compositional parameters were similar than in Figure S19.

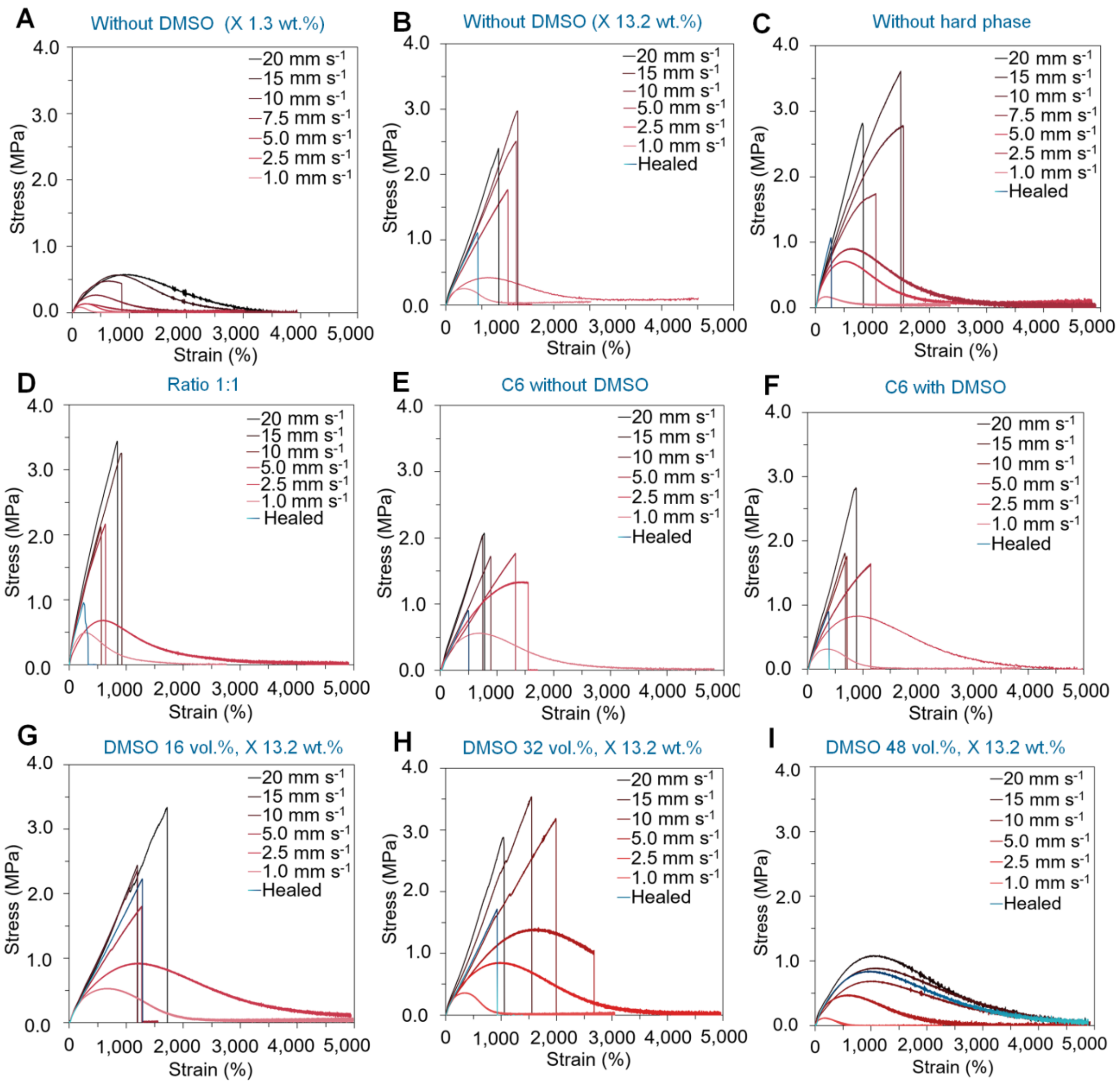

Figure S25. (a-i) Stress-strain curves for elastomers.

(**a-i**) Tensile properties as a function of extension rate for pristine conductors with varied composition. Multiphase conductors (a-b) without DMSO, (c) without hard phase, (d) the ratio was 1:1, (**e-f**) with C6 elastomer composition, and (**g-i**) with varied DMSO content. The other compositional parameters were kept constant: weight of the first solution 59 wt.% and mixing time of the third solution 15 minutes. All other compositions (excluding the bottom row) show poor self-healability in comparison to the best compositions. The self-healing times were ≈120 seconds after the cut-surface alignment.

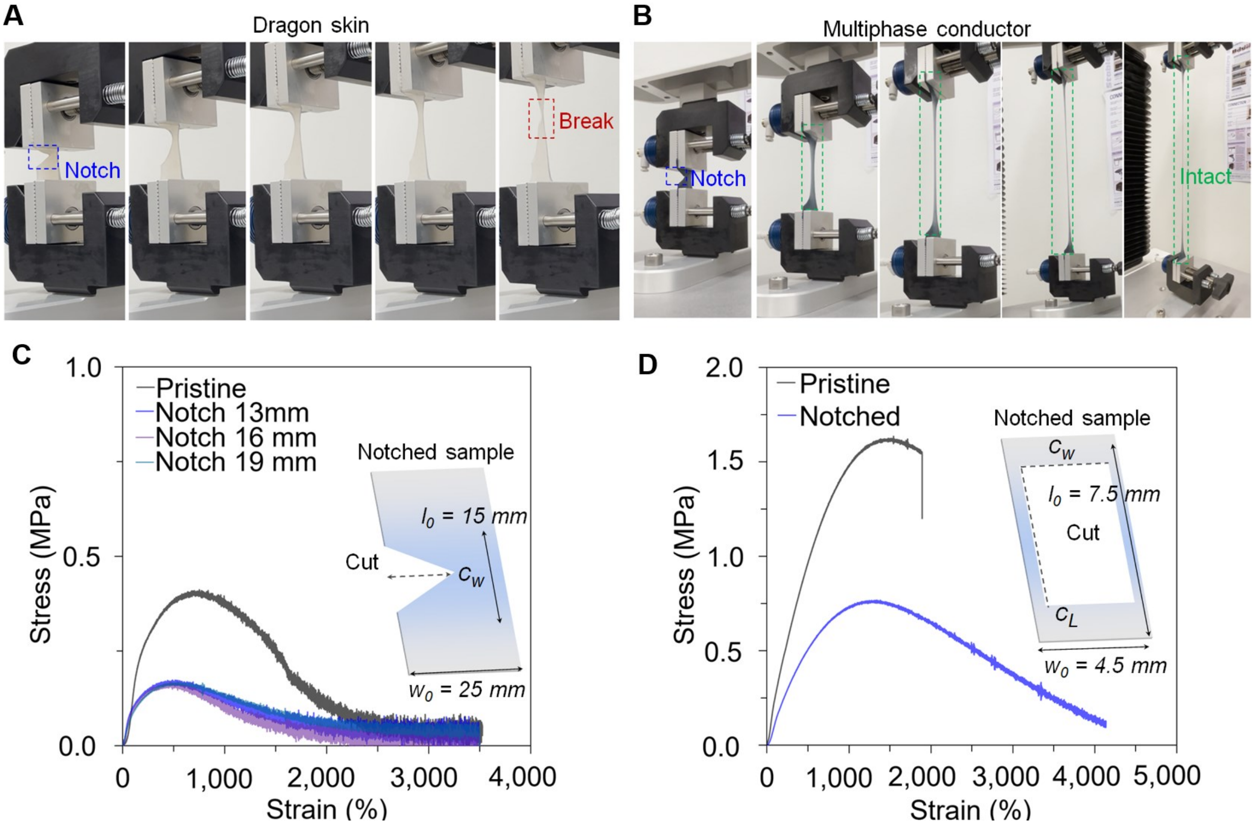

Fig. S26. Stress-strain curves for notched elastomers.

(**a**) Elongating of notched (crack depth (c_w) = 13 mm) Dragon Skin™ elastomer (Smooth-On, Inc.) (width (w_0) = 25 mm) at slow strain rate of 50%s^{-1}. (**b**) Elongating of notched (c_w = 13 mm) multiphase conductor (length (l_0) = 15 mm, w_0 = 25 mm) at strain rate of 50%s^{-1}. (**c**), Stress strain curves for a pristine and notched conductors (c_w = 13, 16, and 19 mm; l_0 = 15 mm, w_0 = 25 mm) with strain rate of 50%s^{-1}. (**d**) Stress-strain curves for a pristine and notched (crack depth (c_w) = 4 mm and crack length (c_l) = 7 mm) at strain rate of 142%s^{-1} after heating to 70 °C. The removed area was approximately 7.0 mm x 4.0 mm (≈90% of the area of the sample). The fracture strain (ε_c) for the conductor was independent of the shape and size of the notch or cut. The notched Dragon Skin™ elastomer fractured at ε_c = 280% which was significantly lower than un-notched sample ($\varepsilon_c \approx$ 1,000%). The flaw sensitivity of the Dragon Skin™ elastomer is typical for also many other commercial elastomer substrates, including Sylgard 184™ PDMS, Ecoflex™ silicone rubber, or VytaFlex™ urethane rubber.

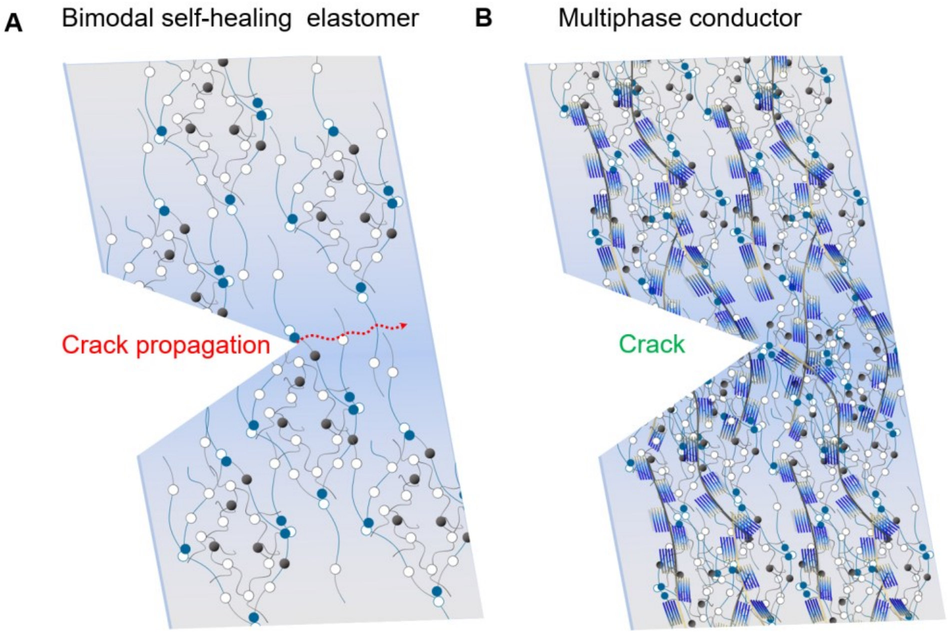

Figure S27. Schematic illustration of (a) crack propagation in notched bimodal self-healing elastomer and (b) flaw insensitivity of the notched multiphase conductor.

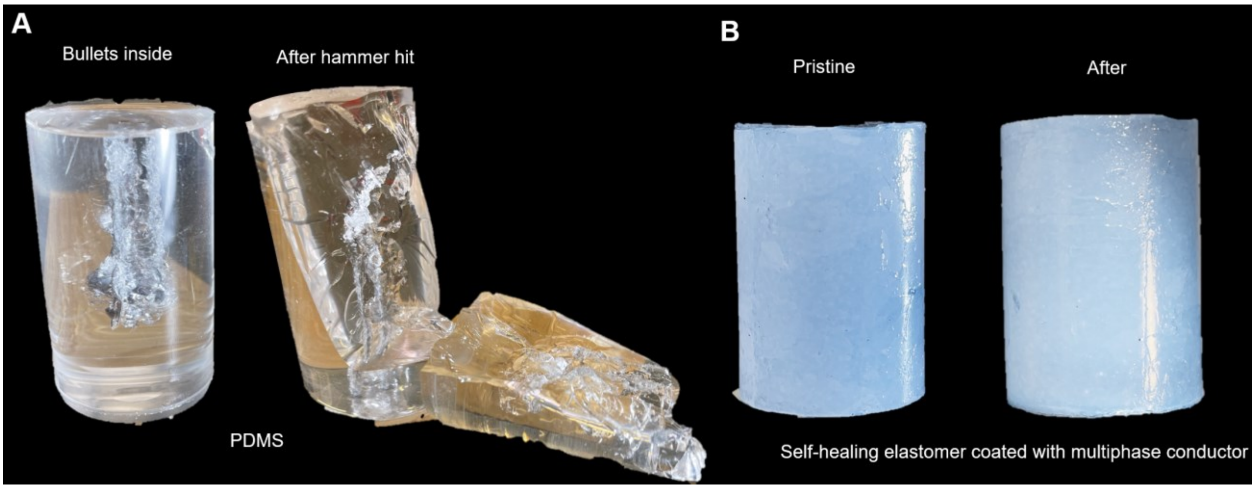

Figure S28. Cylinder-shaped samples after shooting and striking with hammer.
(a) Photographs of Sylgard 184™ PDMS elastomer after shooting with air riffle (left) and after striking with a hammer (right). The samples easily break into pieces after shooting thrice and striking twice. (**b**) Photographs of the pristine self-healing elastomer coated with multiphase conductor film (left) and after shooting and striking with the hammer multiple times (right). There are no visible damages to the cylinder-shaped elastomer that has conductive coating.

Table S1. List of references for Figure S18.

Data	Reference
**1**	Liu, X., Lu, C., Wu, X., Zhang, X., Self-healing strain sensors based on nanostructure supramolecular conductive elastomers. J. Mater. Chem. A, 2017, 5, 9824-9832. https://doi.org/10.1039/C7TA02416A
**2**	Wang, H., Yang, Y., Zhang, M., Wang, Q., Xia, K., Yin, Z., Wie, Y., Ji, Y., Zhang, Y., Electricity-Triggered Self-Healing of Conductive and Thermostable Vitrimer Enabled by Paving Aligned Carbon Nanotubes. ACS Applied Mater. Interfaces, 2020, 12, 12, 14315-14322. https://doi.org/10.1021/acsami.9b21949
**3**	Qin, J., Lin, F., Hubble, D., Wang, Y., Li, Y., Murphy, I. A., Jang, S.-H., Yang, J., Jen, A. K.-Y., Tuning self-healing properties of stiff, ion-conductive polymers. J. Mater. Chem. A, 2019, 7, 6773-6783. https://doi.org/10.1039/C8TA11353J
**4**	Lei, Z., Wu, P. A highly transparent and ultra-stretchable conductor with stable conductivity during large deformation. Nat Commun 10, 3429 (2019). https://doi.org/10.1038/s41467-019-11364-w
**5**	Lei, Z., Wu, P. A supramolecular biomimetic skin combining a wide spectrum of mechanical properties and multiple sensory capabilities. Nat Commun 9, 1134 (2018). https://doi.org/10.1038/s41467-018-03456-w
**6**	Zhang, Q., Chen, G., Li, R., Lin, L., He, M., Mechanically tough yet self-healing transparent conductive elastomers obtained using a synergic dual cross-linking strategy. Polym. Chem., 2021, 12, 2016-2023. https://doi.org/10.1039/D0PY01760D
**7**	Lai, Y.-C., Wu, H.-M., Lin, H.-C., Chang, C.-L., Chou, H.-H., Hsiao, Y.-C., Wu, Y.-C., Entirely, Intrinsically, and Autonomously Self-Healable, Highly Transparent, and Superstretchable Triboelectric Nanogenerator for Personal Power Sources and Self-Powered Electronic Skins. Adv. Funct. Mater., vol. 40, iss. 40, 1904626, 2019. https://doi.org/10.1002/adfm.201904626
**8**	Sun, L., Huang, H., Ding, Q. et al. Highly Transparent, Stretchable, and Self-Healable Ionogel for Multifunctional Sensors, Triboelectric Nanogenerator, and Wearable Fibrous Electronics. Adv. Fiber Mater. (2021). https://doi.org/10.1007/s42765-021-00086-8
**9**	Xu, J., Wang, H., Du, X., Cheng, X., Du, Z., Wang, H., Self-healing, anti-freezing and highly stretchable polyurethane ionogel as ionic skin for wireless strain sensing. Chemical Engineering Journal, vol. 426, 130724 (2021). https://doi.org/10.1016/j.cej.2021.130724
**10**	Zhang, K., Sun, J., Song, J., Gao, C., Wang, Z., Song, C., Wu, Y., Liu, Y., Self-Healing Ti3C2 MXene/PDMS Supramolecular Elastomers Based on Small Biomolecules Modification for Wearable Sensors. ACS Applied Mater. Interfaces, 2020, 12, 40, 45306-45314. https://doi.org/10.1021/acsami.0c13653
**11**	Wang, X., Li, X., Zhao, L., Li, M., Li, Y., Yang, W., Ren, J., Polypyrrole-doped conductive self-healing multifunctional composite hydrogels with a dual crosslinked network. Soft Matter, 2021, 17, 8363-8372. https://doi.org/10.1039/D1SM00682G
**12**	Hou, K.-X., Zhao, S.-P., Wang, D.-P., Zhao, P.-C., Li, C.-H., Zuo, J.-L., A Puncture-Resistant and Self-Healing Conductive Gel for Multifunctional Electronic Skin. Adv. Funct. Mater., 2107006 (2021). https://doi.org/10.1002/adfm.202107006
**13**	Zheng, H., Lin, N., He, Y., Zuo, B., Self-Healing, Self-Adhesive Silk Fibroin Conductive Hydrogel as a Flexible Strain Sensor. ACS Applied Mater. Interfaces, 2021, 13, 33, 40013-40031. https://doi.org/10.1021/acsami.1c08395
**14**	Parida, K., Thangavel, G., Cai, G. et al. Extremely stretchable and self-healing conductor based on thermoplastic elastomer for all-three-dimensional printed triboelectric nanogenerator. Nat Commun 10, 2158 (2019). https://doi.org/10.1038/s41467-019-10061-y
**15**	Lu, Y., Liu, Z., Yan, H., Peng, Q., Wang, R., Barkey, M. E., Jeon, J.-W., Wujcik, E. K., Ultrastretchable Conductive Polymer Complex as a Strain Sensor with a Repeatable Autonomous

	Self-Healing Ability. ACS Applied Mater. Interfaces, 2019, 11, 22, 20453-20464. https://doi.org/10.1021/acsami.9b05464
**16**	Xun, X., Zhang, Z., Zhao, X., Zhao, B., Gao, F., Kang, Z., Liao, Q., Zhang, Y., Highly Robust and Self-Powered Electronic Skin Based on Tough Conductive Self-Healing Elastomer. ACS Nano 2020, 14, 7, 9066-9072. https://doi.org/10.1021/acsnano.0c04158
**17**	Chen, J., Peng, Q., Thundat, T., Zeng, H., Stretchable, Injectable, and Self-Healing Conductive Hydrogel Enabled by Multiple Hydrogen Bonding toward Wearable Electronics. Chem. Mater., 2019, 31, 12, 4553-4563. https://doi.org/10.1021/acs.chemmater.9b01239
**18**	Qu, X., Niu, W., Wang, R., Li, Z., Guo, Y., Liu, X., Sun, J., Solid-state and liquid-free elastomeric ionic conductors with autonomous self-healing ability. Mater. Horiz., 2020, 7, 2994-3004. https://doi.org/10.1039/D0MH01230K
**19**	Zhou, Z., Qian, C., Yuan, W., Self-healing, anti-freezing, adhesive and remoldable hydrogel sensor with ion-liquid metal dual conductivity for biomimetic skin. Composites Science and Technology, vol. 203, 108608 (2021). https://doi.org/10.1016/j.compscitech.2020.108608
**20**	Shin, S.-H., Lee, W., Kim, S.-M., Lee, M., Koo, J. M., Hwang, S. Y., Oh, D. X., Park, J., Ion-conductive self-healing hydrogels based on an interpenetrating polymer network for a multimodal sensor. Chemical Engineering Journal, vol. 371, p. 452-460 (2019). https://doi.org/10.1016/j.cej.2019.04.077
**21**	Yan, S., Zhang,G., Jiang, H., Li, F., Zhang, L., Xia, Y., Wang, Z., Wu, Y., Li, H., Highly Stretchable Room-Temperature Self-Healing Conductors Based on Wrinkled Graphene Films for Flexible Electronics. ACS Applied Mater. Interfaces, 2019, 11, 11, 10736-10744. https://doi.org/10.1021/acsami.9b00274
**22**	Yan, S., Zhang, G., Jin, X., Jiang, H., Li, F., Zhang, L., Wang, Z., Fan, L., Li, H., Rapid room-temperature self-healing conductive nanocomposites based on naturally dried graphene aerogels. J. Mater. Chem. C, 2018, 6, 10184-10191. https://doi.org/10.1039/C8TC03692F
**23**	Han, Y., Wu, X., Zhang, X., Lu, C., Archimedean Spiral Inspired Conductive Supramolecular Elastomer with Rapid Electrical and Mechanical Self-Healing Capability for Sensor Application. Adv. Mater. Technologies, vol. 4, iss. 2, 1800424 (2019). https://doi.org/10.1002/admt.201800424
**24**	Ye., G., Song, Z., Yu, T., Tan, Q., Zhang, Y., Chen, T., He, C., Jin, L., Liu, N., Dynamic Ag–N Bond Enhanced Stretchable Conductor for Transparent and Self-Healing Electronic Skin. ACS Applied Mater. Interfaces, 2020, 12, 1, 1486-1494. https://doi.org/10.1021/acsami.9b17354
**25**	Shi, Y., Wang, M., Ma, C., Wang, Y., Li, X., Yu, G., A Conductive Self-Healing Hybrid Gel Enabled by Metal–Ligand Supramolecule and Nanostructured Conductive Polymer. Nano Lett., 2015, 15, 9, 6276-6281. https://doi.org/10.1021/acs.nanolett.5b03069
**26**	Liu, X., Su, G., Guo, Q., Lu, C., Zhou, T., Zhou, C., Zhang, X., Hierarchically Structured Self-Healing Sensors with Tunable Positive/Negative Piezoresistivity. Adv. Funct. Mater., vol. 28, iss. 15, 1706658 (2018). https://doi.org/10.1002/adfm.201706658
**27**	Cao, Y., Morrissey, T. G., Acome, E., Wong, A. B. M., Keplinger, C., Wang, C., A Transparent, Self-Healing, Highly Stretchable Ionic Conductor. Adv. Mater., vol. 29, iss. 10, 1605099 (2017). https://doi.org/10.1002/adma.201605099
**28**	Jia, Z., Zeng, Y., Tang, P., Gan, D., Xing, W., Hou, Y., Wang, K., Xie, C., Lu, X., Conductive, Tough, Transparent, and Self-Healing Hydrogels Based on Catechol–Metal Ion Dual Self-Catalysis. Chem. Mater., 2019, 31, 15, 5625-5632. https://doi.org/10.1021/acs.chemmater.9b01498
**29**	Shi, P., Wang, Y., Tiju, W. W., Zhang, C., Liu, T., Highly Stretchable, Fast Self-Healing, and Waterproof Fluorinated Copolymer Ionogels with Selectively Enriched Ionic Liquids for Human-Motion Detection. ACS Applied Mater. Interfaces, 2021, 13, 41, 49358-49368. https://doi.org/10.1021/acsami.1c16081
**30**	Lee, J., Tan, W. M., Parida, K., Thangavel, G., Park, S. A., Park, T., Lee, P. S., Water-Processable, Stretchable, Self-Healable, Thermally Stable, and Transparent Ionic Conductors for Actuators and Sensors. Adv. Mater., vol. 32, iss. 7, 1906679 (2020). https://doi.org/10.1002/adma.201906679

**31**	Song, P., Qin, H., Gao, HL. et al. Self-healing and superstretchable conductors from hierarchical nanowire assemblies. Nat Commun 9, 2786 (2018). https://doi.org/10.1038/s41467-018-05238-w
**32**	Deng, Z., Guo, Y., Zhao, X., Ma, P. X., Guo, B., Multifunctional Stimuli-Responsive Hydrogels with Self-Healing, High Conductivity, and Rapid Recovery through Host–Guest Interactions. Chem. Mater., 2018, 30, 5, 1729-1742. https://doi.org/10.1021/acs.chemmater.8b00008
**33**	Li, R., Fan, T., Chen, G., Zhang, K., Su, B., Tian, J., He, M., Autonomous Self-Healing, Antifreezing, and Transparent Conductive Elastomers. Chem. Mater., 2020, 32, 2, 874-881. https://doi.org/10.1021/acs.chemmater.9b04592
**34**	Rong, Q., Lei, W., Chen, L., Yin, Y., Zhou, J., Liu, M., Anti-freezing, Conductive Self-healing Organohydrogels with Stable Strain-Sensitivity at Subzero Temperatures. Angewandte Chemie, vol. 56, iss. 45, p. 14159-14163. https://doi.org/10.1002/anie.201708614
**35**	Lu, C., Wang, C., Wang, J., Yong, W., Chu, F., Integration of hydrogen bonding interaction and Schiff-base chemistry toward self-healing, anti-freezing, and conductive elastomer. Chemical Engineering Journal, vol. 425, 130652 (2021). https://doi.org/10.1016/j.cej.2021.130652
**36**	Xu, L., Huang, Z., Deng, Z., Du, Z., Sun, T. L., Guo, Z.-H., Yue, K. A Transparent, Highly Stretchable, Solvent-Resistant, Recyclable Multifunctional Ionogel with Underwater Self-Healing and Adhesion for Reliable Strain Sensors. Adv. Mater., 2105306 (2021). https://doi.org/10.1002/adma.202105306
**37**	Zhang, Y., Li, M., Qin, B., Chen, L., Liu, Y., Zhang, X., Wang, C., Highly Transparent, Underwater Self-Healing, and Ionic Conductive Elastomer Based on Multivalent Ion–Dipole Interactions. Chem. Mater., 2020, 32, 15, 6310-6317. https://doi.org/10.1021/acs.chemmater.0c00096
**38**	Cao, Y., Tan, Y.J., Li, S. et al. Self-healing electronic skins for aquatic environments. Nat Electron 2, 75–82 (2019). https://doi.org/10.1038/s41928-019-0206-5
**39**	Li, R., Chen, G., Fan, T., Zhang, K., He, M., Transparent conductive elastomers with excellent autonomous self-healing capability in harsh organic solvent environments. J. Mater. Chem. A., 2020, 8, 5056-5061. https://doi.org/10.1039/D0TA00050G
**40**	Jung, D., Lim, C., Shim, H. J., et al. Highly Conductive and elastic nanomembrane for skin electronics. Science, vol. 373, iss. 6558, pp. 1022-1026. https://doi.org/10.1126/science.abh4357
**41**	Wang, Y., Zhu, C., Pfattner, R., et al. A highly stretchable, transparent, and conductive polymer. Science Advances, vol. 3, iss. 3. https://doi.org/10.1126/sciadv.1602076

Table S2. Measured and theoretical Young's moduli.

Measured and theoretical Young's moduli for multiphase conductors with varied insulating to conducting phase ratios and individual phases, and volume fractions.

Ratio	Measured Young's modulus	Volume fraction		Measured Young's modulus			Theoretical Young's modulus
	E_M *(MPa)*	λ	φ	E_1 *(MPa)*	E_2 *(MPa)*	E_3 *(MPa)*	E_T *(MPa)*
2:1	0.160	0.1529	0.9678	1.3	2.0	0.01	0.201
3:1	0.154	0.1516	0.9781	1.3	2.0	0.01	0.199
4:1	0.135	0.1518	0.9835	1.3	2.0	0.01	0.197
5:1	0.153	0.1504	0.9868	1.3	2.0	0.01	0.196
6:1	0.145	0.1502	0.9889	1.3	2.0	0.01	0.196
7:1	0.140	0.1450	0.9905	1.3	2.0	0.01	0.196
8:1	0.138	0.1498	0.9917	1.3	2.0	0.01	0.195
9:1	0.131	0.1497	0.9926	1.3	2.0	0.01	0.195
10:1	0.136	0.1496	0.9933	1.3	2.0	0.01	0.195

Table S3. Mechanical and self-healing properties.

Mechanical and self-healing properties for multiphase conductors with varied composition. For example, 1-1PEC7-16D-X indicates that an insulating to conducting phase ratio was 1:1, self-healing insulating elastomer composition was C7 (reported in our previous work, see reference [13] in the manuscript), DMSO content was 16 vol.%, and X-100 content was 1.3 wt.%. The value inside the brackets indicates a longer mixing time of the third solution (60 minutes). Other parameters were kept constant: weight of the first solution 59 wt.% and mixing time of the third solution 15 minutes.

**Composition**	**Mechanical properties**				**Self-healing properties**	
	E (MPa)	σ_{max} (MPa)	ε_{break} (%)	U_T (MJm^{-3})	$U_{T\text{-healed}}$ (MJm^{-3})	Efficiency (%)
1-1PEC7-16D-X	0.44	2.10	600	6.6	1.7	25.9
2-1PEC7-0D-X13	0.30	2.51	980	12.9	2.0	11.2
2-1PEC6-0D-X13	0.21	1.72	890	7.7	2.2	29.8
2-1PEC7-0D-X1.3	0.19	0.48	870	3.2	-	-
2-1PEC7-16D-X13	0.21	2.34	2340	33.9	39.8	115.0
2-1-PEC7-16D-X13 (60 min)	0.36	2.75	1540	24.7	6.1	24.6
2-1PEC6-16D-X	0.26	1.93	780	9.2	2.2	23.2
2-1PEC7-32D-X	0.22	3.20	2090	21.0	9.6	45.5
2-1PEC7-48D-X	0.19	0.88	4910	18.9	18.3	96.3
3-1PEC7-16D-X	0.21	0.90	2980	22.6	19.9	88.3
4-1PEC7-16D-X	0.13	0.87	4340	28.1	32.9	118.9
5-1PEC7-16D-X	0.18	1.07	4960	27.7	17.8	64.4
6-1PEC7-16D-X	0.19	1.52	2220	24.8	26.7	107.7
7-1PEC7-16D-X	0.10	0.90	4950	29.0	25.9	89.4
8-1PEC7-16D-X	0.17	1.15	2490	21.9	5.7	26.1
9-1PEC7-16D-X	0.22	1.54	2380	26.2	31.4	120.1
10-1PEC7-16D-X	0.15	0.64	2940	14.3	14.6	102.1

Notes: Mechanical properties were measured with 10 mm s^{-1} strain rate in ambient conditions after the cross-linking.

E = Young's modulus (expressed in MPa), σ_{max} = ultimate stress (expressed in MPa), ε_{break} = elongation at break (expressed in %), U_T= toughness (i.e., area under stress-strain curve; expressed in MJm^{-3}). Self-healing efficiency expressed as a ratio of recovered toughness to toughness of a pristine specimen in the same conditions.

Values expressed as mean (n ≥ 3).

Table S4. Self-healing properties.

Self-healing properties for multiphase conductors with varied X-100 content. The other compositional parameters were kept constant: DMSO content 16 vol.%, weight of the first solution 59 wt.%, and mixing time 15 minutes.

Composition	Condition	Toughness (MJ m^{-3})	Self-healing efficiency (%)
2-1PEC7-16D-X1.3	Pristine ambient	13.39 ± 3.15	-
	Pristine cold	33.31 ± 1.69	-
	Pristine underwater	7.16 ± 0.58	-
	Ambient (≈20 °C, 25RT%)	14.13 ± 2.47	105.5
	Vacuum (≈85% vacuum)	9.84 ± 2.33	73.5
	Cold (-19 °C)	33.82 ± 1.50	101.5
	Cold, vacuum (-19 °C, ≈85% vacuum)	31.16 ± 4.57	93.6
	Underwater (20 °C)	7.27 ± 0.96	101.5
	Supercooled NaCI water (-19 °C)	6.77 ± 0.35	93.2
	Alkaline (pH 11)	3.14 ± 0.22	43.8
	Acidic (pH 2.5)	3.89 ± 0.41	54.3
2-1PEC7-16D-X13	Pristine ambient	34.64 ± 0.64	-
	Pristine cold	18.79 ± 4.32	-
	Pristine underwater	7.02 ± 1.33	-
	Ambient (≈20 °C, 25RT%)	39.84 ± 8.81	115.0
	Vacuum (≈85% vacuum)	11.20 ± 3.87	32.3
	Cold (-19 °C)	10.31 ± 0.18	54.9
	Cold, vacuum (-19 °C, ≈85% vacuum)	22.36 ± 4.53	119.0
	Underwater (20 °C)	5.07 ± 0.52	72.2
	Supercooled NaCI water (-19 °C)	3.61 ± 0.87	51.4
	Alkaline (pH 11)	6.22 ± 0.89	88.7
	Acidic (pH 2.5)	7.57 ± 1.76	107.8

Notes: Self-healing properties were compared to pristine samples in similar condition (ambient, cold, or underwater) due to effect of temperature and/or humidity to the tensile properties. Toughness was expressed as a mean ± SD (n ≥ 3). Self-healing times were approximately 120 seconds in dry conditions and 60 seconds in wet conditions.
